# The genome sequence of *Hirschfeldia incana*, a new Brassicaceae model to improve photosynthetic light‐use efficiency

**DOI:** 10.1111/tpj.16005

**Published:** 2022-11-07

**Authors:** Francesco Garassino, Raúl Y. Wijfjes, René Boesten, Francisca Reyes Marquez, Frank F. M. Becker, Vittoria Clapero, Iris van den Hatert, Rens Holmer, M. Eric Schranz, Jeremy Harbinson, Dick de Ridder, Sandra Smit, Mark G. M. Aarts

**Affiliations:** ^1^ Laboratory of Genetics Wageningen University & Research Wageningen Netherlands; ^2^ Bioinformatics Group Wageningen University & Research Wageningen Netherlands; ^3^ Biosystematics Group Wageningen University & Research Wageningen Netherlands; ^4^ Laboratory of Biophysics Wageningen University & Research Wageningen Netherlands; ^5^ Present address: Faculty of Biology Ludwig Maximilian University of Munich Munich Germany; ^6^ Present address: Max Planck Institute for Molecular Plant Physiology Golm Germany

**Keywords:** photosynthesis, Brassicaceae, *Hirschfeldia incana*, model species, high light‐use efficiency, high‐production agriculture, copy number variation

## Abstract

Photosynthesis is a key process in sustaining plant and human life. Improving the photosynthetic capacity of agricultural crops is an attractive means to increase their yields. While the core mechanisms of photosynthesis are highly conserved in C_3_ plants, these mechanisms are very flexible, allowing considerable diversity in photosynthetic properties. Among this diversity is the maintenance of high photosynthetic light‐use efficiency at high irradiance as identified in a small number of exceptional C_3_ species. *Hirschfeldia incana*, a member of the Brassicaceae family, is such an exceptional species, and because it is easy to grow, it is an excellent model for studying the genetic and physiological basis of this trait. Here, we present a reference genome of *H. incana* and confirm its high photosynthetic light‐use efficiency. While *H. incana* has the highest photosynthetic rates found so far in the Brassicaceae, the light‐saturated assimilation rates of closely related *Brassica rapa* and *Brassica nigra* are also high. The *H. incana* genome has extensively diversified from that of *B. rapa* and *B. nigra* through large chromosomal rearrangements, species‐specific transposon activity, and differential retention of duplicated genes. Duplicated genes in *H. incana*, *B. rapa*, and *B. nigra* that are involved in photosynthesis and/or photoprotection show a positive correlation between copy number and gene expression, providing leads into the mechanisms underlying the high photosynthetic efficiency of these species. Our work demonstrates that the *H. incana* genome serves as a valuable resource for studying the evolution of high photosynthetic light‐use efficiency and enhancing photosynthetic rates in crop species.

## Significance Statement

*Hirschfeldia incana* is a diploid, wild species, closely related to *Brassica* crops, which displays remarkably high photosynthesis rates at high irradiance, a desirable trait to improve in crops to increase yield. We propose *H. incana* as an attractive high‐photosynthesis model species and present the reference genome sequence as a valuable resource for studying the evolution, genetics, and physiology of high photosynthetic light‐use efficiency at high irradiances.

## INTRODUCTION

Photosynthesis is the biophysical and biochemical process that sustains most life on planet Earth. The most common form of photosynthesis, oxygenic photosynthesis, uses solar energy to convert inorganic carbon dioxide (CO_2_) to organic carbon, typically represented as a carbohydrate, releasing molecular oxygen (O_2_) from water in the process. Terrestrial plants provide by far the most conspicuous example of oxygenic photosynthesis (referred to as photosynthesis from now on for brevity) and are responsible for about 50% of the primary production of oxygen in the biosphere, with marine production by eukaryotic algae and cyanobacteria comprising the other 50%. Agriculture depends on primary production by plants, so expanding our knowledge of photosynthesis is crucial if we are to meet many of the pressing global challenges faced by humankind.

One of these challenges is the need to substantially increase the yield of agricultural crops to meet the increasing demand not only for food and fodder, but also for fibres and similar plant products, and organic precursors for the chemical industry as it transitions away from fossil carbon sources. A major yield‐related trait is the conversion efficiency of absorbed solar irradiance to biomass (*ɛ*_*c*_; Long et al., 2006), a parameter which is strongly influenced by the light‐use efficiency of photosynthesis. As light intensity, or irradiance, increases, the photosynthetic light‐use efficiency of leaves and other photosynthetic organs decreases, which leads ultimately to light saturation of photosynthesis (Genty & Harbinson, 1996; Gitelson et al., 2015; Gu et al., 2017; Monneveux et al., 2003; Murchie et al., 1999; Turner et al., 2003). Once light saturation is reached, any additional light will not lead to a further increase in the photosynthetic rate and may even be detrimental to photosynthesis. The threshold for light saturation generally lies far below the maximum level of irradiance experienced in the field or greenhouse (Zhu et al., 2010) and for most C_3_ crops this light saturation phenomenon is an aspect of their photosynthesis which remains to be increased in order to increase yield. Improving the photosynthetic light‐use efficiency of crop plants thus paves the way towards increasing their *ɛ*_*c*_ and ultimately their yield (Flood et al., 2011; Furbank et al., 2019; Lawson et al., 2012; von Caemmerer & Evans, 2010; Zhu et al., 2010), as recently shown in soybean (*Glycine max*; De Souza et al., 2022).

The means with which to reduce the loss of photosynthetic light‐use efficiency in crop plants with increasing irradiance already exists in nature. Most temperate‐zone crop species, alongside tropical crops species like rice (*Oryza sativa*), make use of the C_3_ photosynthetic pathway, which is the original and ancestral photosynthetic pathway in higher plants, with the alternative CAM and C_4_ pathways having evolved as an adaptation to heat and drought, and low CO_2_ levels. Due to several issues associated with the C_3_ pathway compared to the C_4_ pathway, the maximum photosynthesis rates commonly observed among C_3_ species are generally lower than those of C_4_ ones. Although the core mechanisms of photosynthesis are highly conserved (Leister, 2019; Shi et al., 2005), natural variation in photosynthesis rates has been observed for major crops such as wheat (*Triticum aestivum*; Driever et al., 2014), rice (Gu et al., 2012; Gu et al., 2014), maize (*Zea mays*; Strigens et al., 2013), soybean (Gilbert et al., 2011), and sorghum (*Sorghum bicolor*; Ortiz et al., 2017), as well as for the model species *Arabidopsis thaliana* (van Rooijen et al., 2015; van Rooijen et al., 2017). Much higher photosynthesis rates can be expected in species that are more ecologically specialised (van Bezouw et al., 2019). Exceptionally high light‐use efficiencies (and high assimilation rates) at high irradiance have been found previously in species growing in the Sonoran Desert, such as *Amaranthus palmeri*, *Chylismia claviformis*, *Eremalche rotundifolia*, and *Palafoxia linearis* (Ehleringer, 1985; Werk et al., 1983). Although data collected on these species provided clues about the anatomical and physiological basis of their high photosynthesis rates (Gibson, 1998; Werk et al., 1983), a comprehensive ecophysiological explanation of their phenotypes is still missing.

To understand the physiological and genetic basis of this more efficient photosynthesis at high irradiance, a suitable model species is needed. To date, of the handful of species showing high light‐use efficiency that have been described (Ehleringer, 1985; Werk et al., 1983), none would qualify as a model species due to a combination of complex genetics and difficulties in growing in laboratory conditions (e.g. difficult seed germination). Taking inspiration from *A. thaliana*, an attractive model species for high light‐use efficiency would need to be easily grown in either regular irradiance (typically up to 600 μmol m^−2^ sec^−1^) and high‐light laboratory conditions; have a high‐quality reference genome; be a diploid species capable of producing a large number of progeny (hundreds of seeds from a single mother plant) with a short generation time; germinate easily and have easily stored seed; and allow for both inbreeding and outcrossing (Koornneef & Meinke, 2010; Somerville & Koornneef, 2002).

*Hirschfeldia incana* (L.) Lagr.‐Foss. is an excellent candidate that fulfils these requirements. *Hirschfeldia incana* is a thermophilous and nitrophilous annual species native to the Mediterranean basin and the Middle East, but currently widespread in most warm‐temperate regions of the world (Siemens, 2011). It is generally self‐incompatible and thus allogamous, but a degree of self‐compatibility has been observed in natural populations (Lee et al., 2004). Although it makes use of the C_3_ pathway, *H. incana* has a very high photosynthesis rate at high irradiance (Canvin et al., 1980), much higher than that of the C_3_ crop species wheat (Driever et al., 2014) and rice (Gu et al., 2012), more in the range of C_4_ species (Crafts‐Brandner & Salvucci, 2002; Leakey et al., 2006). Besides its exceptional physiological properties, *H. incana* is also an attractive model species for practical and genetic reasons. It shows fast and sustained growth in laboratory conditions and is a member of the Brassiceae tribe within the well‐studied Brassicaceae family, allowing the use of many genetic and genomic resources developed for the model species *A. thaliana* and its close relatives *Brassica rapa* (Belser et al., 2018; Choi et al., 2007; Kim et al., 2009; The Brassica rapa Genome Sequencing Project Consortium, 2011; Zhang et al., 2018), *Brassica nigra* (Paritosh et al., 2020; Perumal et al., 2020), *Brassica oleracea* (Belser et al., 2018; Liu et al., 2014; Wang et al., 2011), and *Brassica napus* (Bancroft et al., 2011; Chalhoub et al., 2014). Yet, *H. incana* has received little attention from the research community so far, being recognised mainly as a possible lead (Pb) hyperaccumulator (Auguy et al., 2013; Auguy et al., 2016; Fahr et al., 2015) and for the ecological implications of its occurrence as a weed (Darmency & Fleury, 2000; Lee et al., 2004; Liu et al., 2013; Mira et al., 2019; Sánchez‐Yélamo, 2009).

Here we present a genomic assembly and gene set of *H. incana*. We expect these data to lay the foundation for studying photosynthetic light‐use efficiency and improving this trait in crop species, through a process of candidate gene identification followed by phenotypic validation using genetic modification and/or gene editing. First, we directly compare the photosynthetic rate of *H. incana* at high irradiance to that of the Brassicaceae species *B. rapa*, *B. nigra*, and *A. thaliana* to affirm its high light‐use efficiency. Second, we characterise how the *H. incana* genome differs from that of other members of the Brassicaceae family, specifically focusing on differences in numbers of gene copies. Finally, we report on whether such differences translate to differential expression of genes expected to mediate high light‐use efficiency. Our work demonstrates how the genome assembly of *H. incana* serves as a valuable resource to elucidate the genetic basis of high photosynthetic performance and for studying the evolution of this trait in the Brassicaceae family.

## RESULTS

### *Hirschfeldia incana* has an exceptionally high rate of photosynthesis

**Figure 1:**
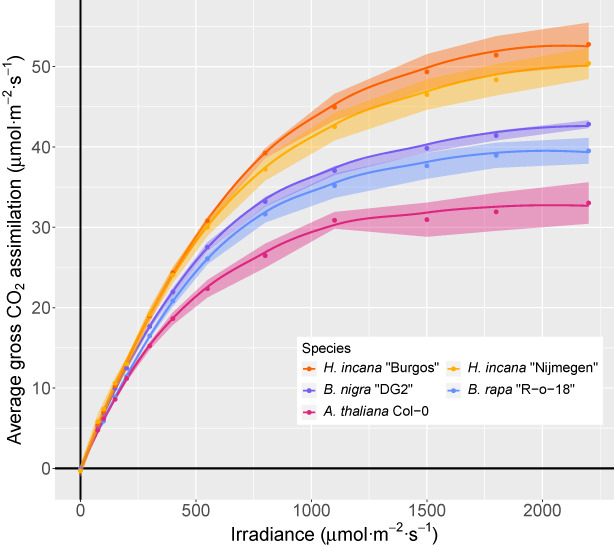
Two *H. incana* genotypes have a higher gross CO_2_ assimilation at high irradiance than genotypes of close relatives. Light–response curves for *H. incana*, *B. rapa*, *B. nigra*, and *A. thaliana* accessions adapted to high levels of irradiance. Each point represents the mean gross CO_2_ assimilation value of three (*B. rapa*) or four leaves coming from independent plants. Ribbons represent the standard error of the means. The lines indicate trends in gross assimilation for the various species and were obtained via LOESS smoothing.

High photosynthesis rates have been reported for *H. incana* in 1980 (Canvin et al., 1980). We performed new measurements in order to compare the performance of *H. incana* with that of close relatives and the well‐established model species *A. thaliana* (Figure 1, Table S1). Gross CO_2_ assimilation rates differed significantly between these species (Table S2). The two *H. incana* accessions had the highest average gross CO_2_ assimilation rates above an irradiance of 550 μmol m^−2^ sec^−1^ (photosynthetically active radiation), although only ‘Burgos’ had a statistically significant higher rate than the other species (Table S3). Gross assimilation rates are independent of CO_2_ release by mitochondrial respiration and therefore a better indication of photosynthetic capacity than net photosynthesis rates; however, net photosynthesis rates showed a similar trend, but with larger differences between the two *H. incana* genotypes (Figure S1, Table S3), attributed to differences in rates of daytime dark respiration (*R*_*d*_, Table S4).

### A reference genome of *H. incana*

We assembled a scaffold‐level reference genome of *H. incana* based on one genotype of the ‘Nijmegen’ accession. *H. incana* is a predominantly self‐incompatible species, but ‘Nijmegen’ produces, nonetheless, some 100 seeds per plant upon self‐pollination, which is much more than ‘Burgos’, so inbreeding is possible. Inbred genotypes are expected to be much more homozygous than open‐pollinated genotypes, which is preferred for genome sequencing. Therefore, the ‘Nijmegen’ accession was inbred for six generations, prior to whole‐genome sequencing. Its haploid genome size was estimated to be 487 Mb, based on flow cytometry (Table S5). This estimate is smaller than the previously reported genome size estimates of *B. rapa* (529 Mb) and *B. nigra* (632 Mb) (Johnston et al., 2005). Chromosome counts from root tip squashes showed seven pairs of chromosomes (2 *n* = 14) (Figure S2), consistent with previous reports (Anderson & Warwick, 1999; Siemens, 2011).

We generated DNA sequencing data consisting of 56 Gb of PacBio long reads (115‐fold genome coverage, based on the genome size estimate), 46 Gb of 10X Genomics synthetic long reads (94‐fold coverage, referred to as ‘10X’ from now on for brevity), and 33 Gb of Illumina paired‐end short reads (68‐fold coverage). In addition, we generated 7.5 Gb of RNA sequencing (RNA‐seq) data from leaf tissue for annotation purposes. Summary statistics and accession numbers can be found in Table S6. A k‐mer analysis of Illumina data resulted in a haploid genome size estimate of 325 Mb, with a low level of heterozygosity (1.2%).

Using a hybrid assembly strategy, we produced a nuclear genome assembly of 399 Mb of sequence in 384 scaffolds with an N50 of 5.1 Mb (Table 1, see Table S7 for the full report generated by QUAST; Gurevich et al., 2013). The assembly size is slightly larger than the genome size estimated from Illumina read k‐mers (325 Mb), but smaller than the typical overestimate (Sun et al., 2018) based on flow cytometry (487 Mb). Besides the nuclear genome, we assembled the mitochondrial and chloroplast genomes of *H. incana* into single sequences of 253 and 153 kb, and annotated the latter. The chloroplast assembly is typical for a Brassicaceae species, as it is nearly identical to chloroplast assemblies of *A. thaliana*, *B. rapa*, and *B. nigra* in terms of length and number of annotated genes (Table S8), and thus very useful for phylogenetic comparison of *H. incana* with other Brassicaceae.

**Table 1:** Genomic properties of assemblies generated of *H. incana* ‘Nijmegen’ (this study), *B. rapa* Chiifu 401‐42 (Zhang et al., 2018), and *B. nigra* Ni100 (Perumal et al., 2020)

	*H. incana*	*B. rapa*	*B. nigra*
Technologies	PacBio, 10X, Illumina paired‐end	PacBio, BioNano, Hi‐C, Illumina mate‐pair	Nanopore, Hi‐C, genetic mapping
Size (Mb)	398.5	353.1	506
# scaffolds	384	1301	58
N50 (Mb)	5.1	4.4	60.8
Gaps (kb)	0.54	0.40	13
GC content (%)	36.2	36.8	37.0
Complete BUSCO assembly (%)	96.2	97.7	97.0
Complete BUSCO annotation (single/duplicated) (%)	95.1 (80.2/14.9)	97.2 (84.2/13.0)	97.2 (81.9/15.3)
# protein‐coding genes	32 313	46 250	59 852
# protein‐coding transcripts	38 706	46 250	59 852
Repeat content (%)	49.4	37.5	54.0
Full‐length LTR‐RTs (%)	25.3	29.2	41.8

The nuclear genome assembly is near complete and structurally consistent with the underlying read data of *H. incana* ‘Nijmegen’ (Table S9). The high mapping rate of Illumina and 10X reads (>93%) suggests completeness, while the lower mapping rate of PacBio reads (81.5%) suggests some misassemblies or missing regions, likely repeats. The high mapping rate of RNA‐seq reads (93.6%) also shows the gene space is near complete. We estimated the base‐level error rate of the assembly to be 1 per 50 kb at most, based on variant calling using the mapped reads, resulting in 8374 and 4166 homozygous variants from the Illumina and 10X read alignments, respectively.

We have annotated 32 313 gene models and 38 706 transcripts in the *H. incana* assembly (Table 1). This is a conservative annotation, based on filtering 64 546 initial gene models resulting from *ab initio* protein alignment and RNA‐seq‐based predictions. Our filtering approach is more stringent than those used to generate the *B. rapa* and *B. nigra* annotations, which explains why we report a lower number of genes and transcripts for *H. incana* (Table 1) than for both *Brassica* species. The annotation is expected to cover the large majority of the *H. incana* gene space. It contains 95.1% of 1440 single‐copy orthologues (BUSCOs) conserved in the Embryophyta plant clade, comparable to the percentages found for *B. rapa* and *B. nigra* (both 97.2%) (Table 1). The ratio of single to multiple copies is similar to that of *B. rapa* and *B. nigra* (Table 1), suggesting that the 14.9% of the BUSCOs present in multiple copies are true gene duplications shared by several species of the Brassiceae tribe. We additionally evaluated the completeness of the annotation by aligning protein sequences of *B. rapa* to the assembly and determining overlap between protein alignments and annotated genes. Out of the 37 387 protein alignments (81.7%), 30 552 corroborate the annotation, as they completely or partially overlap with an annotated protein‐coding gene. Of the protein alignments, 2570 (6.9%) completely or partially overlap with an annotated repeat, suggesting that the aligned *B. rapa* proteins correspond to transposable elements (TEs). The remainder of the *B. rapa* proteins completely or partially overlaps with gene models that were filtered (3945, 10.6%) or does not overlap with any annotated element at all (320, 0.9%), indicating a small number of genes that are potentially missing from the annotation. Based on these observations, we conclude that the *H. incana* assembly is mostly contiguous, correct, and complete, making it a solid foundation for comparative analyses with other Brassicaceae.

### The genome of *H. incana* extensively diversified from that of *B. rapa* and *B. nigra*

We utilised our assembly to explore the genomic divergence between *H. incana*, *B. rapa*, and *B. nigra*, all members of the same Brassiceae tribe. A substantial degree of divergence is expected between the three species due to different processes of post‐polyploid diploidization, i.e. the process in which polyploid genomes get extensively rearranged as they return to a diploid state (Mandáková & Lysak, 2018), following the ancient two‐step genome triplication event shared by all Brassiceae (He et al., 2021; Lysak et al., 2005; The Brassica rapa Genome Sequencing Project Consortium, 2011). Part of this divergence may have facilitated the evolution of the exceptionally high rate of photosynthesis at high irradiance in *H. incana*.

**Figure 2:**
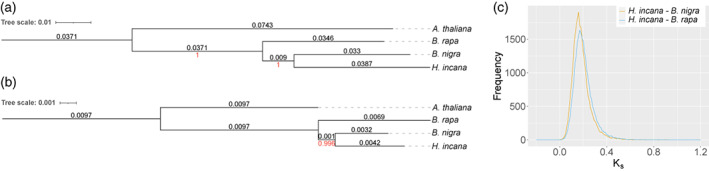
*Hirschfeldia incana* is more closely related to *B. nigra* than *B. rapa*. (a,b) Phylogenetic trees of *H. incana*, *B. rapa*, *B. nigra*, and *A. thaliana* (outgroup), based on nuclear (a) and chloroplast (b) genes. Branch lengths (black) and bootstrap values (red) are displayed above and below each branch, respectively. (c) Distributions of the rates of synonymous substitutions between 25 127 and 26 137 orthologous gene pairs of *H. incana* and *B. rapa* and between *H. incana* and *B. nigra*, respectively. Both distributions show a single peak corresponding to speciation events with an estimated time of 11.6 (*H. incana* and *B. rapa*) and 10.4 (*H. incana* and *B. nigra*) mya.

We first assessed the phylogenetic relationship between *H. incana*, *B. rapa*, and *B. nigra* by constructing phylogenetic trees based on homologous nuclear and chloroplast genes, using *A. thaliana* as the outgroup. Both trees are congruent with each other and suggest that *H. incana* is more closely related to *B. nigra* than *B. rapa* (Figure 2a,b). This is corroborated by the median rate of synonymous substitutions between the syntenic orthologues (*K*_*s*_) of the three species, which correspond to speciation events with an estimated time of 10.4 (*H. incana* and *B. nigra*) and 11.6 (*H. incana* and *B. rapa*) million years ago (mya) (Figure 2c) as obtained by dividing the median *K*_*s*_ of each curve by the rate of 8.22 × 10^−9^ synonymous substitutions per year established for Brassicaceae species (Beilstein et al., 2010). Our results are consistent with a previous phylogenetic analysis based on four intergenic chloroplast regions (Arias & Pires, 2012), but slightly differ from a more recently constructed phylogeny of the Brassicaceae, which was only based on 113 nuclear genes (Huang et al., 2016), while we consider many more.

**Figure 3:**
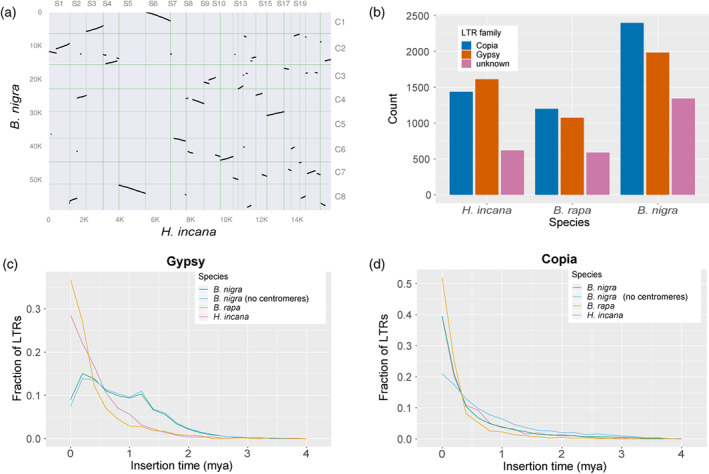
The genome of *H. incana* extensively diversified from that of *B. rapa* and *B. nigra*. (a) Orthologous syntenic blocks between the genomes of *H. incana* and *B. nigra*. Dots indicate pairs of syntenic orthologs that are found in the same order in both genomes according to sequence positions. Only the 20 largest scaffolds of *H. incana* (43.6% of the assembly) are shown for clarity. Axis labels correspond to the total number of genes annotated on the sequences (left and bottom) and identifiers of the scaffolds (top) or chromosomes (right). A dot plot visualising orthologous syntenic blocks between *H. incana* and *B. rapa*, showing similar patterns, is found in Figure S3. (b) Frequency distribution of long terminal repeat retrotransposon (LTR‐RT) families. LTR‐RTs are classified as unknown if they contained elements of both Gypsy and Copia sequences and could thus not be reliably assigned to either of these families. (c) Frequency polygon (bin width = 0.2 mya) of the insertion times of Gypsy elements. (d) Frequency polygon (bin width = 0.2 mya) of insertion times of Copia elements.

We determined rearrangements between the genomes of *H. incana* and *B. rapa* and between those of *H. incana* and *B. nigra* by comparing the order of syntenic orthologues between their assemblies. On a small scale, most genomic regions of *H. incana* are syntenic (not rearranged) with *B. rapa* and *B. nigra*, as 77.7% and 81.0% of the genes of *H. incana* could be clustered in collinear blocks containing a minimum of four orthologous pairs of *H. incana* and *B. rapa* and of *H. incana* and *B. nigra*, respectively. Gene order is less conserved when comparing larger blocks, indicating several rearrangements between the 20 largest scaffolds of *H. incana* (covering 43.6% of the assembly) and the chromosomes of the other two species (Figures 3a and S3). For example, the two largest scaffolds of the *H. incana* assembly both contain inversions and/or translocations relative to their homologous chromosomes in *B. rapa* and *B. nigra*. A similar pattern of rearrangements of small collinear blocks was observed between the genomes of *B. rapa* and *B. nigra* in previous work (He et al., 2021).

We further examined genomic differentiation between the three species by comparing their TE content. Of the assembly of *H. incana*, 49.4% consists of repetitive elements (Table 1), of which most are long terminal repeat retrotransposons (LTR‐RTs) (25.3% of the genome). These numbers are consistent with previous work that investigated the repeat content of the *H. incana* genome using genome skimming, and which reported a repeat content of 46.5% and an LTR‐RT content of 31.6% (Beric et al., 2021). We specifically focused our analyses on LTR‐RTs, as LTR‐RT expansion and contraction has been previously identified as a major driver of genomic differentiation between Brassiceae (C. Xu et al., 2018), even between different ecotypes of the same species (Cai et al., 2020). The composition of LTR‐RTs in the *H. incana* assembly differs from that of the *B. rapa* and *B. nigra* assembly, as the majority of LTR‐RTs consist of Gypsy elements in *H. incana*, consistent with earlier work (Beric et al., 2021), while Copia retrotransposons form the majority of LTR‐RTs in the others (Figure 3b). Furthermore, the estimated insertion times of LTR‐RTs vary between the three assemblies, as Gypsy and Copia elements in *H. incana* and *B. rapa* are predicted to have proliferated recently (<1 mya) (Figure 3c,d), while Gypsy elements in *B. nigra* show a more varied distribution of insertion times (Figure 3c). A possible explanation of this shift could be that the *B. nigra* assembly was generated using longer reads than those used for the assemblies of *H. incana* and *B. rapa*, enabling it to capture a larger proportion of the centromeric regions, but we found no evidence that this introduced a bias towards longer insertion times of Gypsy elements (Figure 3c).

Taken together, the breakdown of genomic synteny and divergence of LTR‐RT content indicates that the genome of *H. incana* extensively diversified from that of *B. rapa* and *B. nigra* following their shared genome triplication event.

### Gene copy number expansion may contribute to high photosynthetic rates

Genomic differentiation can result in species‐specific gains and losses of genes, and these may explain the differences in photosynthetic light‐use efficiency between *H. incana*, *B. rapa*, and *B. nigra*. Given that the three species all share the same ancient genome triplication event (He et al., 2021; Schranz et al., 2006), it is reasonable to assume that most differences originated through differential retention of duplicated genes, particularly those located in genomic blocks showing evidence of extensive fractionation since that event (He et al., 2021). We investigated gene copy number variation between the three species by clustering their annotated protein‐coding genes with those of five other Brassicaceae species within (*Raphanus raphanistrum* and *Raphanus sativus*) and outside (*Aethionema arabicum*, *A. thaliana*, and *Sisymbrium irio*) the Brassiceae tribe into homology groups. The inclusion of *A. thaliana* allowed us to use its extensive genomic resources to functionally annotate the genes of the other species. The other four species were included to put the analysis into a broader phylogenetic context. *Aethionema arabicum* is part of the Aethionema tribe, which diverged from the core group of the Brassicaceae family, thus allowing us to identify highly conserved genes. *Sisymbrium irio* is part of a different tribe than *A. thaliana* (Sisymbrieae) that is more closely related to the Brassiceae tribe (Huang et al., 2016), but did not undergo the ancient genome triplication. *Raphanus raphanistrum* and *R. sativus* are part of the *Raphanus* genus within the Brassiceae tribe and thus represent another set of species that underwent the genome triplication event shared by the whole tribe.

Our analysis resulted in 20 331 groups containing at least one *H. incana* gene (Table S10). The composition of the homology groups agrees with the currently established phylogeny of the Brassicaceae (Huang et al., 2016) as groups containing *H. incana* genes share the least number of genes with *A. arabicum* (58.2%) and the greatest number of genes with species that are part of the Brassiceae tribe (86.3–95.6%). *Hirschfeldia incana* has a low fraction of species‐specific homology groups (3.4%) compared to the seven other species, which can be attributed to the stringent filtering of the predicted gene models.

**Figure 4:**
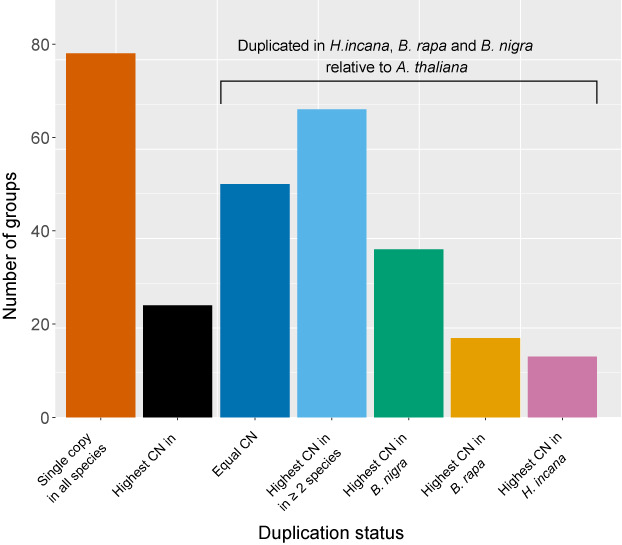
*Hirschfeldia incana* retained fewer duplicated copies of photosynthesis‐associated genes than *B. rapa* and *B. nigra*. Bars show counts of homology groups containing genes associated with photosynthesis with different distributions of copy numbers (CNs) in the four species (260 groups in total). For groups that contain a higher number of copies in *H. incana*, *B. rapa*, and *B. nigra* than in *A. thaliana*, it has been indicated whether the same number of copies is found in all three species (equal CN), whether there are two or more species that contain a higher number of copies than the other(s) (highest CN in at least two species), or whether there is a single species containing the highest number of copies.

We focused on a subset of 15 097 groups containing at least one gene of *A. thaliana* and one of *H. incana*, as these could be extensively annotated through the transfer of Gene Ontology (GO) terms from *A. thaliana* genes to their respective groups. Consistent with the expectation that most genes quickly return to single‐copy status following a whole‐genome duplication event (Z. Li et al., 2016), 70.2% of these groups contain a single gene of both *A. thaliana* and *H. incana*. Focusing on groups containing *A. thaliana* genes involved in photosynthesis (260 in total, Table S11), most contain a higher number of genes of *H. incana*, *B. rapa*, and *B. nigra* compared to *A. thaliana* (Figure 4), consistent with the relatively higher photosynthetic light‐use efficiency of *H. incana*, *B. rapa*, and *B. nigra* (Figure 1). That *H. incana* should have the highest efficiency of all the four species is not apparent from the gene copy number data because for most groups of genes, *H. incana* contains the same or a lower number of copies relative to *B. rapa* and *B. nigra*. This is not a result of our conservative filtering approach, as we explicitly retained putative photosynthesis‐related genes during our filtering procedure (Methods S1). Besides photosynthesis‐related genes, we also analysed copy numbers of a more general set. We found that 4901 homology groups contain genes of which the copy number in *H. incana* is higher than in *A. thaliana* and equal to or higher than those of *B. rapa* and *B. nigra* (Table S12). We estimate that 74.5% of the duplicated gene pairs in *H. incana* (16 788 of the 22 535 analysed pairs) were duplicated through whole‐genome duplication, 1.8% through tandem duplication, and the remaining 23.6% through another mode of duplication. Given that the increased photosynthetic light‐use efficiency of *H. incana* relative to *A. thaliana*, *B. rapa*, and *B. nigra* is particularly pronounced at high levels of irradiance (Figure 1), genes annotated with GO terms associated with photosynthesis and/or photoprotection are of particular interest. The 4901 homology groups contain ample examples of such genes (Table S12), although the groups are not significantly enriched for any GO term specifically linked to photosynthesis and/or photoprotection (Table S13).

**Figure 5:**
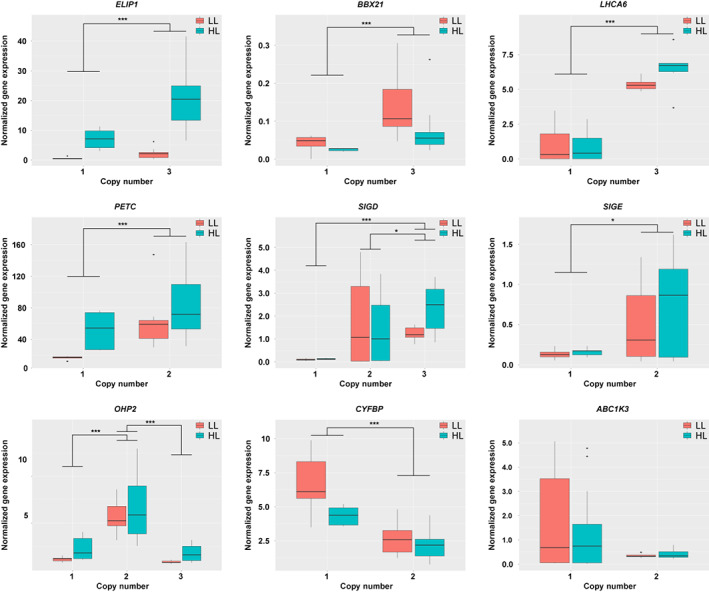
Copy numbers of photosynthesis‐ and photoprotection‐associated genes correlate with expression level. Boxplots depict gene expression levels of *A. thaliana*, *B. rapa*, *B. nigra*, and *H. incana* grown in 200 μmol m^−2^ sec^−1^ and 1500 μmol m^−2^ sec^−1^. Gene expression levels were normalised against *H. incana* grown at 200 μmol m^−2^ sec^−1^ and subsequently grouped per gene copy number. Titles of graphs indicate gene names based on the *A. thaliana* gene nomenclature. *P < 0.05; **P < 0.01; ***P < 0.001.

As gene copy number variation can considerably affect expression levels (Żmieńko et al., 2014), we hypothesised that retained copy number expansions of photosynthesis‐ and photoprotection‐associated genes in *H. incana*, *B. rapa*, and *B. nigra* may contribute to the high photosynthetic capacities of these species (Figure 1). We therefore measured gene expression levels of nine genes for which there is inter‐species copy number variation in two contrasting light conditions (200 μmol m^−2^ sec^−1^ and 1800 μmol m^−2^ sec^−1^), selecting genes with a function related to photosynthesis and/or photoprotection (Table 2). *Arabidopsis thaliana*, the species with the lowest photosynthesis rates measured in this study, contains a single copy of each of the tested genes. For six genes, we observed a statistically significant positive correlation between gene expression level and gene copy number (Figure 5), with species showing higher or equal expression with an increasing number of copies (Figure S4). No such correlation was observed for the remaining three genes.

**Table 2:** Genes with inter‐specific copy number variation of which expression was measured. All genes are annotated to a function in photosynthesis and/or photoprotection

Gene name	Full gene name		Copy number		
		*A. thaliana*	*B. rapa*	*B. nigra*	*H. incana*
*LHCA6*	*PHOTOSYSTEM I LIGHT* *HARVESTING COMPLEX* *GENE 6* (Peng et al., 2009)	1	1	1	3
*ELIP1*	*EARLY LIGHT* *INDUCED PROTEIN* (Hutin et al., 2003)	1	3	3	3
*SIGE/SIG5*	*SIGMA FACTOR 5* (Tsunoyama et al., 2004)	1	2	2	2
*SIGD/SIG4*	*SIGMA FACTOR 4* (Favory et al., 2005)	1	2	2	3
*BBX21*	*B‐BOX DOMAIN PROTEIN* *21* (Crocco et al., 2018)	1	3	3	3
*PETC*	*PHOTOSYNTHETIC* *ELECTRON TRANSFER C* (Maiwald et al., 2003)	1	2	2	2
*ABC1K3*	*ABC1‐LIKE KINASE 3* (Martinis et al., 2013)	1	1	1	2
*OHP2*	*ONE‐HELIX PROTEIN 2* (Y. Li et al., 2019)	1	2	2	3
*CYFBP*	*CYTOSOLIC FRUCTOSE‐1,6‐BISPHOSPHATASE* (S.‐K. Lee et al., 2008)	1	2	2	2

To test if the observed differences in gene expression are due to photosynthesis‐related genes being more frequently more highly expressed in general in *B. rapa*, *B. nigra*, and *H. incana* compared to *A. thaliana*, we included nine additional genes in our experiment that are present in a single copy in all four species and involved in similar processes as the multi‐copy genes. Although we find species‐specific differences in expression for this set of genes, no consistently higher gene expression levels are found in *B. rapa*, *B. nigra*, and *H. incana* compared to *A. thaliana* (Figure S5). Overall, our analyses suggest that the increased copy numbers of photosynthesis‐ and photoprotection‐associated genes in *H. incana*, *B. rapa*, and *B. nigra* relative to *A. thaliana* may contribute to their high photosynthetic efficiency, although this effect appears to not be specific to a particular species or level of irradiance.

## DISCUSSION

In this study, we generated a reference genome of *H. incana* to establish this species as a model for exceptional photosynthetic light‐use efficiency at high irradiance. We find substantial differences in light‐use efficiency, genomic structure, and gene content between *H. incana* and its close relatives. We discuss these results in terms of how they contributed to the evolution of the remarkable phenotype of *H. incana*.

Our results show an even higher photosynthetic light‐use efficiency at high irradiance than previously reported for *H. incana* (Canvin et al., 1980), with photosynthesis rates varying marginally between both accessions. Examination of a wider set of *H. incana* accessions may identify genotypes with larger differences in photosynthesis rates, which would allow a quantitative genetic approach to identify alleles conferring high photosynthesis rates. Our measurements imply that the photosynthesis rates of this C_3_ species are higher than those of the C_4_ crop maize (Crafts‐Brandner & Salvucci, 2002; Leakey et al., 2006) and almost two times higher than those typically reported from key cereal crop species with C_3_ photosynthetic metabolism, such as wheat (Driever et al., 2014) and rice (Gu et al., 2012). Furthermore, these rates are higher than those of the closely related Brassicaceae species *B. rapa* and *B. nigra* and the more distantly related *A. thaliana*. The photosynthesis rates we measured in *B. rapa* are also higher than previously reported (Pleban et al., 2018; Taylor et al., 2020). Although the rates presented in this study were obtained from plants grown in controlled, favourable conditions and thus could be an overestimation of rates in natural environments, the magnitude of the differences suggests that the *H. incana* genome holds essential information for the improvement of photosynthetic light‐use efficiency in crops.

The reference genome of *H. incana* generated in this study provides the means to elucidate the genetic basis of this plant's exceptional rate of photosynthesis and how it evolved in this species. We estimate that *H. incana* diverged 11.6 and 10.4 mya from *B. rapa* and *B. nigra*, respectively, consistent with an earlier study that used a smaller set of nuclear genes (Huang et al., 2016). These time points are close to the reported time at which *B. rapa* and *B. nigra* (Perumal et al., 2020) diverged from each other (11.5 mya) and the time at which the whole Brassicaceae family underwent a rapid radiation event (Franzke et al., 2009). This event may have been mediated by the expansion of grass‐dominated ecosystems in the region inhabited by Brassicaceae family members at that time, which created new open habitats that favoured rapid diversification (Franzke et al., 2009). This expansion of grasslands is thought to have been driven by decreasing atmospheric CO_2_ levels and increasing aridity, which favoured the displacement of the then dominant C_3_ plants by C_4_ grasses (Edwards et al., 2010). We argue that climatic changes also drove the evolution of the high photosynthetic rates observed in *H. incana*; grassland, i.e. non‐forested ecosystems, may have provided the ephemeral niches with high irradiances that favoured the evolution of high photosynthetic rates. Species with high photosynthetic rates are currently found in Mediterranean and desert ecosystems (Ehleringer, 1985; Werk et al., 1983). The evolution of high rates of C_3_ photosynthesis could therefore have paralleled the expansion of the C_4_ photosynthesis pathway as an adaptation to low CO_2_ levels and drought.

Our analyses suggest that the genome of *H. incana* extensively differentiated from the genomes of *B. rapa* and *B. nigra* since their time of divergence, through large genomic arrangements and differences in LTR‐RT content. Previous analyses of natural *A. thaliana* accessions indicated that specific LTR‐RT families show increased rates of proliferation in response to particular types of environmental stress (Baduel et al., 2021), which may explain the species‐specific amplification of Gypsy elements that we observed in *H. incana*. Such elements may have been retained because this particular LTR‐RT family generally inserts outside of exons (Baduel et al., 2021). We hypothesise that the differences in LTR‐RT content between *H. incana*, *B. rapa*, and *B. nigra* were caused in part by Gypsy elements being less efficiently purged from the genome of *B. nigra* than from the others. An increased rate of LTR‐RT removal, based on the ratio of solo LTRs to intact LTR‐RTs, has also been observed in *B. rapa* relative to *B. oleracea* and it was speculated that this is caused by the increased rate of genetic recombination in the former (Zhao et al., 2013). Given that a similar negative correlation between local recombination rate and LTR‐RT content was found in rice (Tian et al., 2009), soybean (Du et al., 2012), and eukaryotes in general (Kent et al., 2017), the differences in predicted insertion times of Gypsy elements in *H. incana*, *B. rapa*, and *B. nigra* observed in this study may thus reflect different rates of genetic recombination in the three species. While it has been suggested that changes in recombination rate can be adaptive, there is little empirical evidence that supports this (Ritz et al., 2017). It would therefore be interesting to directly measure genome‐wide rates of recombination of *H. incana*, *B. rapa*, and *B. nigra* and explore whether these are correlated with their rates of photosynthesis.

Further comparative analyses between the genomes of *H. incana*, *B. rapa*, *B. nigra*, and *A. thaliana* revealed numerous species‐specific gains and losses of genes. For dosage‐sensitive genes, such as those involved in transcriptional regulation, differences may not necessarily reflect adaptive selection. This category of genes was found to be consistently retained in multiple copies following polyploidy events across the Brassicaceae (Mandáková et al., 2017) and a wide group of angiosperms (Z. Li et al., 2016), which is hypothesised to be due to dosage constraints (Edger & Pires, 2009). Differences in copy number of such genes may thus reflect different rates of relaxation of dosage balance constraints and subsequent loss of duplicates through time, which is a neutral process.

On the other hand, there is reason to believe that gene duplications contributed to the evolution of the high light‐use efficiency of *H. incana*. Gene duplications have been identified as important drivers of plant evolution and differences in gene copy number between species are often enriched for adaptive evolutionary traits (Dassanayake et al., 2011; Oh et al., 2013; Rizzon et al., 2006; Suryawanshi et al., 2016). Moreover, reverse transcriptase quantitative PCR (RT‐qPCR) analysis of nine duplicated genes associated with photosynthesis and/or photoprotection showed that the expression levels of six of them correlate with gene copy number. In contrast, nine photosynthetic genes present in a single copy in all species did not show significantly increased expression in *H. incana*, *B. nigra*, and *B. rapa* compared to *A. thaliana*, indicating that photosynthetic genes are not overexpressed in the former three species in general. This supports a putative role for gene duplications in mediating the high light‐use efficiency achieved by *H. incana*, *B. nigra*, and *B. rapa*.

The most striking genes of which copy number correlated with gene expression are *LHCA6* and *ELIP1*, involved in response to high light and having the highest expression in *H. incana* growing under high light (Figure S4). *LHCA6* encodes a light‐harvesting complex I (LHCI) protein of photosystem I (PSI) that together with LHCA5 is required to form a full‐size NAD(P)H dehydrogenase (NDH)‐PSI supercomplex (Peng et al., 2009). Higher expression of *LHCA6* might help the formation of the NDH‐PSI complex, thought to help stabilise NDH under high‐irradiance conditions. In turn, NDH has proposed roles supporting the Calvin–Benson cycle's activity (Harbinson et al., 2022) and photoprotection by preventing overreduction at high light intensities (Munekage et al., 2004). *ELIP1* encodes a protein with a proposed role in photoprotection and is associated with high‐light stress (Heddad et al., 2006; Norén et al., 2003; Youssef et al., 2010). Increased expression of this gene is expected to make the photosynthetic apparatus of *H. incana* more resistant to photoinhibition at high levels of irradiance. While the *H. incana* genome harboured the highest number of copies of *LHCA6* compared to the genomes of *A. thaliana*, *B. rapa*, and *B. nigra*, this is not the case for *ELIP1*, for which *H. incana*, *B. nigra*, and *B. rapa* all have three copies as opposed to the single copy of *A. thaliana*. Therefore, although we can propose a role for gene duplications in achieving higher light‐use efficiency, the exact nature of this role still remains unclear as it appears to not be completely dependent on species or light treatment.

While our gene expression analysis provides several promising leads, it only offers a glimpse of what may contribute to the high photosynthetic light‐use efficiency of *H. incana*. Besides the nine genes included in this analysis, we identified many more genes with a high copy number in *H. incana* that warrant further investigations on a whole‐transcriptome level. Such investigations should not be limited to core photosynthetic genes, as *H. incana* can only attain high photosynthetic light‐use efficiency through changes in many other traits that are outside the chloroplast, such as leaf architecture affecting mesophyll conductance to CO_2_, the synthesis of carbohydrates in the cytosol, the transport of carbohydrates from the leaf, the uptake from the soil and the supply of nitrogen and other minerals to the leaf, the abundance and distribution of different leaf pigments, and (photo)respiration. Nor should they include duplicated genes only, as it is striking that *H. incana* shows a higher light‐use efficiency than *B. rapa* and *B. nigra*, though it contains fewer photosynthesis‐related genes than the latter two species. This points towards alternative scenarios in which adaptation of *H. incana* photosynthesis to high levels of irradiance occurred through regulation of expression of one copy of the photosynthesis‐related genes, which relaxed selection on duplicate retention or even encouraged loss of duplicate copies, or through other traits, as described above.

To elucidate the exact genetic mechanisms underlying the high light‐use efficiency of *H. incana*, a natural follow‐up to this study is to perform comparative transcriptomic analyses of leaves of *H. incana*, *B. rapa*, and *B. nigra* under a range of different levels of irradiance and at different developmental stages. Genes that show copy number variation and are differentially expressed between *H. incana* and the latter two species, such as *LHCA6*, would then be prime candidates to further test for potential causality through e.g. knock‐out mutant analysis. As previous work has shown that it is possible to cross distantly related Brassicaceae species (Katche et al., 2019), a useful approach to further pinpoint the causal genes is to establish a genetic mapping population between *H. incana* and a Brassicaceae species with regular light‐use efficiency and perform quantitative trait locus analyses of photosynthetic traits segregating within the population. It would also be useful to expand comparative genome and transcriptome analyses to plant species outside of the Brassicaceae family that show high photosynthetic light‐use efficiency, such as the aforementioned *A. palmeri*, *C. claviformis*, *E. rotundifolia*, and *P. linearis*. Such expanded analyses could be informative, for instance to investigate amino acid substitutions or lateral gene transfer specific to species with high photosynthetic light‐use efficiency. Furthermore, transcriptome data may indicate genes showing differences in expression between such species and those that are less efficient, providing further insight into which genes contribute to the evolution of this trait.

## CONCLUSIONS

*Hirschfeldia incana* has an exceptional rate of photosynthesis at high irradiance. We generated a near‐complete reference genome of this species and found evidence suggesting that its exceptional rate evolved through differential retention of duplicated genes. Taken together, our work provides several promising leads that may explain the high photosynthetic light‐use efficiency of *H. incana*, and we expect the reference genome generated in this study to be a valuable resource for improving this efficiency in crop cultivars.

## MATERIALS AND METHODS

### Plant material

*Hirschfeldia incana* accessions ‘Nijmegen’ and ‘Burgos’ were used. ‘Nijmegen’ is an inbred line (more than six rounds of inbreeding) originally collected in Nijmegen, the Netherlands. Seeds of ‘Burgos’ were originally collected near Burgos, Spain. Furthermore, *B. nigra* accession ‘DG2’, sampled from a natural population near Wageningen, the Netherlands, the *B. rapa* inbred line ‘R‐o‐18’ (Bagheri et al., 2012; Stephenson et al., 2010), and *A. thaliana* accession Col‐0 were used.

### Measurements of photosynthesis rates

Seeds of *H. incana* ‘Nijmegen’, *H. incana* ‘Burgos’, *B. rapa* ‘R‐o‐18’, *B. nigra* ‘DG2’, and *A. thaliana* Col‐0 were sown in 3‐L pots filled with a peat‐based potting mixture. Plants were grown in a climate chamber with a 12/12‐h photoperiod at day and night temperatures of 23°C and 20°C, respectively. Relative humidity and CO_2_ levels were set at 70% and 400 ppm, respectively. The chamber was equipped with high‐output LED light modules (VYPR2p, Fluence by OSRAM). Plants were watered daily with a custom nutrient solution (0.6 mm NH_4_^+^, 3.6 mm K^+^, 2 mm Ca^2+^, 0.91 mm Mg^2+^, 6.2 mm NO_3_^−^, 1.66 mm SO_4_^2−^, 0.5 mm P, 35 μm Fe^3+^, 8 μm Mn^2+^, 5 μm Zn^2+^, 20 μm B, 0.5 μm Cu^2+^, 0.5 μm Mo^4+^). The seeds were germinated at an irradiance of 300 μmol m^−2^ sec^−1^, and the same irradiance was maintained to let seedlings establish. On days 14, 21, and 25 after sowing, the irradiance was raised to 600, 1200, and 1800 μmol m^−2^ sec^−1^, respectively.

The photosynthetic metabolism of young, fully expanded leaves developed under 1800 μmol m^−2^ sec^−1^ of light was measured with a LI‐COR 6400XT portable gas exchange system (LI‐COR Biosciences) equipped with a 2‐cm^2^ fluorescence chamber head. ‘Rapidly’ descending light–response curves were measured between 30 and 35 days after sowing to accommodate differences in growth rates of the different species on one leaf from four *H. incana* ‘Nijmegen’, *H. incana* ‘Burgos’, *B. nigra* ‘DG2’, and *A. thaliana* Col‐0 plants and three *B. rapa* ‘R‐o‐18’ plants. The net assimilation rates of the plants were measured at 13 different levels of irradiance ranging from 0 to 2200 μmol m^−2^ sec^−1^. During measurements, leaf temperature was kept constant at 25°C and the reference CO_2_ concentration was kept at 400 μmol mol^−1^. Water in the reference air flux was regulated in order to achieve vapour pressure deficit values between 0.8 and 1.2 kPa.

Light–response curve parameters (*A*_*max*_: net CO_2_ assimilation at saturating irradiance; *φ*: apparent quantum yield of CO_2_ assimilation; *R*_*d*_: daytime dark respiration rate; and *θ*: curve convexity) were estimated for each species through non‐linear least squares regression of a non‐rectangular hyperbola (Marshall & Biscoe, 1980) with the R package ‘photosynthesis’ (version 2.0.0) (Stinziano et al., 2020). An indication of gross assimilation rates for each species was subsequently generated by adding the daytime dark respiration rate (*R*_*d*_) estimated for each species to the species' net assimilation rates.

Differences in net and gross assimilation rates were tested at each light level of the light–response curve with one‐way analysis of variance (anova) on the ‘genotype’ experimental factor. Pairwise comparisons between the assimilation rates of the different genotypes at each light level were subsequently performed and tested with the Tukey–Kramer extension of Tukey's range test. The *P*‐value threshold for statistical significance was set at α = 0.05.

### Flow cytometry

Leaf samples of the *H. incana* genotypes ‘Burgos’ and ‘Nijmegen’ and *A. thaliana* Col‐0 were analysed for nuclear DNA content by flow cytometry (Plant Cytometry Services B.V., Didam, the Netherlands). Seven, three, and five biological replicates were measured over separate rounds of analysis for *H. incana* ‘Nijmegen’, *H. incana* ‘Burgos’, and *A. thaliana* Col‐0, respectively. Nuclei were extracted from leaf samples following the method by Arumuganathan & Earle, 1991, and stained with 4′,6‐diamidino‐2‐phenylindole (DAPI). The DNA content of nuclei relative to that of the reference species *Monstera deliciosa* was determined on a CyFlow Ploidy Analyser machine (Sysmex Corporation, Kobe, Japan). A haploid flow cytometry estimate of 157 Mb was used for *A. thaliana*, resulting from comparisons of nuclear DNA content of this species and other model organisms (Bennett et al., 2003). Haploid genome size estimates for the *H. incana* genotypes were obtained by multiplying the *H. incana*‐to‐*M. deliciosa* ratio by the haploid *A. thaliana* estimate and dividing this product by the average *A. thaliana*‐to‐*M. deliciosa* ratio.

### Chromosome counting

Root tips (approximately 1 cm long) were collected from young, fast‐growing rootlets of multiple *H. incana* ‘Nijmegen’ plants and pre‐treated for 3 h at room temperature with a 0.2 mm 8‐hydroxyquinoline solution. After pre‐treatment, the 8‐hydroxyquinoline solution was replaced with freshly prepared Carnoy fixative (1:3 [v/v] acetic acid:ethanol solution) and maintained at room temperature for half a day. Root tips were then rinsed with 70% ethanol three times to remove remaining fixative and stored in 70% ethanol at 4°C until further use. Prior to slide preparation, root tips were rinsed twice in Milli‐Q (MQ) water before adding 1:1 solution of a pectolytic enzymatic digestion solution (1% Cellulase from *Trichoderma*, 1% Cytohelicase from *Helix Promatia*, 1% Pecolyase from *Aspergillus japonicus*) and 10 m citric buffer. After incubation at 37°C for 1 h, the enzymatic digestion solution was replaced by MQ water. The digested root tips were spread in 45% acetic acid over microscopy slides on a hot plate set at 45°C, and cells were fixed with freshly prepared Carnoy fixative, dried, and stained with DAPI dissolved in Vectashield mounting medium (Vector Laboratories Inc., Burlingame, US). Slides were imaged with an Axio Imager.Z2 fluorescence optical microscope coupled with an Axiocam 506 microscope camera (Carl Zeiss AG, Oberkochen, Germany) at 63× magnification. Chromosome numbers were counted in metaphase mitotic cells and averaged to obtain the reported number.

### DNA and RNA isolation

Genomic DNA was extracted from *H. incana* ‘Nijmegen’ samples using a protocol modified from Chang et al., 1993. The modifications consisted of adding 300 μL β‐mercaptoethanol to the extraction buffer just before use. We added 0.7% isopropanol to the supernatant instead of 10 m LiCl and then divided the total volume into 1‐ml aliquots for subsequent extractions. The pellet was dissolved in 500 μl of SSTE which was pre‐heated to 50°C before use. The final pellets were dissolved in 50 μl MQ water and then pooled at the end of the extraction process. DNA used for Illumina and 10X Genomics sequencing was extracted from flower material, while leaf material was used for PacBio sequencing, all originating from the same plant.

Total RNA was extracted from leaf material of *H. incana* ‘Nijmegen’ from a different plant than the one used for the DNA isolations with the Direct‐zol RNA mini‐prep kit (Zymo Research, Irvine, CA, USA) according to the company's instructions and then subjected to DNAse (Promega Corporation, Madison, WI, USA) treatment at 37°C for 1 h.

### Generation of sequencing data

Sequencing of total‐cellular DNA of *H. incana* ‘Nijmegen’ was performed by GenomeScan B.V., Leiden. A total of seven SMRT cells were used for sequencing on the Pacific Biosciences Sequel platform. Short‐read Illumina and 10X Genomics libraries with an insert size of approximately 500–700 bp were prepared with the NEBNext Ultra DNA Library Prep kit for Illumina and the 10X Genomics Chromium^TM^ Genome v1 kit, respectively. These libraries were sequenced using an Illumina X10 platform (2 × 151 bp). RNA paired‐end sequencing libraries with an average insert size of 254 bp were prepared using the Illumina TruSeq RNA sample prep kit with polyA mRNA selection and sequenced using an Illumina HiSeq 2500 platform (2 × 125 bp).

### k‐Mer analysis

A histogram of k‐mer frequencies of Illumina reads predicted to be of nuclear origin (Methods S1) was generated using Jellyfish (v2.2.6) (Marçais & Kingsford, 2011) using a k‐mer length of 21. The resulting histogram was provided as input to GenomeScope (v1.0.0) (Vurture et al., 2017) to estimate genome size and heterozygosity.

### Genomic assembly and annotation

The genome assembly and annotation process is more extensively described in Methods S1. In short, we generated an initial assembly based on the PacBio data only with Canu (Koren et al., 2017) and used it to bin the PacBio, 10X, and Illumina reads according to whether they originated from nuclear, organellar, or contaminant DNA. The bins were used to separately assemble the nuclear and organellar genomes, yielding a nuclear assembly consisting of hundreds of contigs and mitochondrial and chloroplast assemblies that were both represented by a single sequence. Nuclear contigs representing alternative haplotypes were removed using purge_dups (Guan et al., 2020), after which ARKS (Coombe et al., 2018) was used to scaffold the remaining contigs using the 10X data. Scaffolds were polished using Arrow (https://github.com/PacificBiosciences/gcpp) and Freebayes (Garrison & Marth, 2012), followed by a manual filtering step to obtain the final nuclear assembly.

Repeats in the assembly were masked using RepeatMasker (Smit et al., 2015) in combination with RepeatModeler2 (Flynn et al., 2020) before starting the annotation procedure. Nuclear genes were annotated using EvidenceModeler (Haas et al., 2008) to generate consensus models of *ab initio* gene predictions, alignments of proteins from closely and distantly related plant species, and transcripts assembled from RNA‐seq data. These models were manually filtered to obtain a final set of protein‐coding genes.

### Used datasets for comparative genome analyses

We mainly focused the comparative genome analyses on *H. incana*, *B. nigra*, and *B. rapa*, three species of the Brassiceae tribe, of which all members underwent an ancient genome triplication (Lysak et al., 2005; The Brassica rapa Genome Sequencing Project Consortium, 2011). For comparative gene analyses, we extended this group with the Brassicaceae species *A. thaliana*, *A. arabicum*, *S. irio*, *R. raphanistrum*, and *R. sativus*. The latter two *Raphanus* species are also part of the Brassiceae tribe. Version numbers and locations of all genomes are listed in Table S14.

### Analysis of pairwise gene synteny and long terminal repeat retrotransposons in *H. incana*, *B. rapa*, and *B. nigra*

Analyses of pairwise gene synteny between scaffolds of *H. incana* and chromosomes of *B. rapa* and *B. nigra* were performed using the JCVI library (https://github.com/tanghaibao/jcvi) (v1.0.5) in Python. Orthologues were identified through all‐versus‐all alignment of genes with LAST (Kiełbasa et al., 2011), retaining reciprocal best hits only (C‐score of at least 0.99). Hits were filtered for tandem duplicates (hits located within 10 genes from each other) and chained using the Python implementation of MCScan (Tang et al., 2008) to obtain collinear blocks containing at least four pairs of syntenic genes. Visualisations of collinearity between genomic assemblies were generated using custom scripts of JCVI.

*K*_*s*_ values of syntenic gene pairs were computed using the ks module of JCVI. Protein sequences of pairs were aligned against each other using MUSCLE (v3.8.1) (Edgar, 2004), after which PAL2NAL (v14) (Suyama et al., 2006) was used to convert protein alignments to nucleotide ones. *K*_*s*_ values for each pair were computed from the nucleotide alignments using the method of Yang & Nielsen, 2000 implemented in PAML (Yang, 2007) (v4.9). Times of divergence between species were estimated by dividing the median of the distributions of their *K*_*s*_ values by the rate of 8.22 × 10^−9^ synonymous substitutions per year that was established for Brassicaceae species based on extrapolation from the ancient triplication event in the Brassica clade (Beilstein et al., 2010).

Putative LTR‐RTs were identified using LTRharvest (v1.6.1) (Ellinghaus et al., 2008) and LTR_finder (v1.1) (Z. Xu & Wang, 2007), after which LTR_retriever (v2.9.0) (Ou & Jiang, 2018) was run with default parameters to filter and combine the output of both tools into a high‐confidence set. LTR_retriever was also used to provide estimates of the insertion time of each LTR‐RT. Parameters of LTRharvest and LTR_finder were set as recommended in the LTR_retriever documentation. Centromeric regions of the *B. nigra* assembly were obtained from Table S21 of the manuscript describing the assembly (Perumal et al., 2020).

### Phylogenetic analysis of *H. incana*, *B. rapa*, and *B. nigra*

The longest isoforms of the nuclear genes of *H. incana*, *B. rapa*, *B. nigra*, and *A. thaliana* (outgroup) were provided to Orthofinder (version 2.3.11) (Emms & Kelly, 2019) to generate phylogenetic species trees. Orthofinder was run using the multiple sequence alignment workflow with default parameters. The same analysis was performed using chloroplast genes. Trees were visualised using iTOL (version 6.3) (Letunic & Bork, 2021).

### Comparative Gene Ontology analysis of eight Brassicaceae species

The longest isoforms of the genes of all eight Brassicaceae species described in the section ‘Used datasets for comparative genome analyses’ were extracted using AGAT (version 0.2.3) (https://github.com/NBISweden/AGAT) and clustered into homology groups using the ‘group’ function of Pantools version 2 (Sheikhizadeh Anari et al., 2018) with a relaxation parameter of 4. Groups were assigned GO slim terms of their associated *A. thaliana* genes (obtained from https://www.arabidopsis.org/download_files/GO_and_PO_Annotations/Gene_Ontology_Annotations/TAIR_GO_slim_categories.txt; last updated on 1 July, 2020) and GO terms assigned to protein domains of associated *H. incana*, *B. rapa*, and *B. nigra* genes using InterProScan (version 5.45–72.0) (Jones et al., 2014) (ran using the Pfam and Panther databases only). GO term enrichment tests were performed using the Fisher exact test and the Benjamini–Hochberg method for multiple testing correction (Benjamini & Hochberg, 1995). *Arabidopsis thaliana* genes were considered to be involved in photosynthesis if they fulfilled one of the following conditions:

- annotated with one of the following GO terms: ‘photosynthesis’, ‘electron transporter, transferring electrons within the cyclic electron transport pathway of photosynthesis activity’, or ‘electron transporter, transferring electrons within the noncyclic electron transport pathway of photosynthesis activity;’
- included in the KEGG pathways ath00195 (Photosynthesis), ath00710 (Carbon fixation in photosynthetic organisms), or ath00196 (Photosynthesis ‐ Antenna Proteins);
- protein products have been assigned the keyword ‘Photosynthesis’ in the Swiss‐Prot database.

The same criteria were used to retain photosynthesis‐related genes of *H. incana* while filtering the gene annotation of the assembly (Methods S1).

### Investigating the mode of duplicated genes in *H. incana*

Dupgen_finder (GitHub commit hash 8001838) (Qiao et al., 2019) was run with default parameters to determine the mode of duplication for duplicated gene pairs in *H. incana*, using the genome of *A. thaliana* as an outgroup to detect pairs duplicated through whole‐genome duplication. Pairs were allowed to be assigned to a single category only. Input files containing alignments of the protein sequences of *H. incana* aligned to themselves and those of *A. thaliana* were prepared using DIAMOND (version 0.9.14) (Buchfink et al., 2015).

### Analysis of gene expression under high and low irradiance

Seeds of *H. incana* ‘Nijmegen’, *B. nigra* ‘DG2’, *B. rapa* ‘R‐o‐18’, and *A. thaliana* Col‐0 were germinated on a peat‐based potting mixture for nine days under an irradiance of 200 μmol m^−2^ sec^−1^. Twelve seedlings per species were then transferred to 2‐L pots filled with a peat‐based potting mixture enriched with perlite and 2.5 g L^−1^ Osmocote® Exact Standard 5‐6M slow‐release fertiliser (ICL Specialty Fertilizers, Geldermalsen, the Netherlands).

Plants were germinated and grown in a climate chamber with a 12/12‐h photoperiod at day and night temperatures of 23°C and 20°C, respectively. Relative humidity and CO_2_ levels were set at 70% and 400 ppm, respectively. The chamber was equipped with high‐output LED light modules (VYPR2p, Fluence by OSRAM, Austin, USA). Six plants per species were assigned to high‐light (HL) treatment (1800 μmol m^−2^ sec^−1^) and the remaining six were assigned to low‐light (LL) treatment (200 μmol m^−2^ sec^−1^). Irradiance uniformity was very high for both HL and LL treatments, with a U2 value of 0.93. Plant positions were randomised across growing areas. Plants were watered with the same custom nutrient solution as the one used in the measurements of photosynthesis rates, daily for LL treatment and twice a day for HL treatment.

Twenty‐eight days after sowing, one young fully adapted leaf from each plant was selected, excised, and snap‐frozen in liquid nitrogen. Leaf samples were crushed with a mortar and pestle cooled with liquid nitrogen and further homogenised with glass beads for 2 min at 30 Hz in an MM300 Mixer Mill (Retsch GmbH, Haan, Germany). Total RNA was extracted with the RNeasy Plant Mini Kit (QIAGEN N.V., Venlo, the Netherlands) according to the manufacturer's instructions and then subjected to a RQ1 DNAse treatment (Promega Corporation, Madison, US) at 37°C for 30 min. We validated the total removal of DNA by means of a no‐reverse transcriptase PCR reaction on all RNA samples. RNA was assessed for purity (A_260_/A_280_) with a Nanodrop 2000 spectrophotometer (Thermo Fisher Scientific Inc., Waltham, US) and for possible RNA degradation by means of visual inspection of the RNA on a 1% agarose gel. cDNA was then synthesised from 2 μg total RNA (measured by a spectrophotometer) with the SensiFAST™ cDNA Synthesis Kit (Meridian Bioscience, Cincinnati, US) according to the manufacturer's instructions.

To examine the expression of both single‐copy and multi‐copy photosynthesis‐ and/or photoprotection‐related genes (Table S15), species‐specific RT‐qPCR primers were designed with the following criteria: the PCR fragment size had to be between 80 and 120 bp, the maximum difference in melting temperature between primers of the same pair had to be 0.5°C, and overall melting temperatures had to be between 58°C and 62°C. Primers were designed to target a region of the gene as similar as possible in all species. Additionally, for multi‐copy genes, the primer pair had to bind to all copies of a particular gene in one species. RT‐qPCR reactions were performed with SYBR green on a CFX96 Real‐Time PCR Detection System (Bio‐Rad Laboratories Inc., Hercules, US). The efficiency of each designed primer set was assessed by means of a standard curve, and only primer sets with efficiencies ranging between 90% and 110% were used. All primer sequences can be found in Table S16.

Gene expression was normalised to the reference genes *ACT2*, *PGK*, *UBQ7*, and *APR* (Joseph et al., 2018; Løvdal & Lillo, 2009) using the ΔCt method (Livak & Schmittgen, 2001). Normalised gene expression values were calculated as 2^−ΔCt^. For the statistical analysis, we performed two‐way anova on the ΔCt values with the copy number and light treatment as grouping variables for the multi‐copy genes and species and light treatment as grouping variables for the single‐copy genes. A Kenward–Roger approximation was used for the degrees of freedom, and a post hoc test was subsequently performed with Tukey's range test, with the significance threshold set at α = 0.05.

## AUTHOR CONTRIBUTIONS

JH and MGMA initiated the research on the genomic basis of high photosynthesis of *H. incana*. MGMA generated the inbred line of *H. incana* ‘Nijmegen’. FG grew plants for the gene expression experiment and performed measurements of photosynthesis rates, flow cytometry experiments, and subsequent analyses of experimental data. FRM performed RNA extractions, cDNA synthesis, and qPCR reactions for the gene expression experiment. FG and VC selected genes and designed primers. RB analysed the results of the gene expression experiment. RB and FB performed DNA and RNA extractions for genome sequencing and assembly. RYW and SS generated strategies for the genome assembly and annotation, which were applied by RYW. RYW and IvdH performed comparative analyses of Brassicaceae genomes. RH was involved in genome annotation. MES helped interpreting the results of the comparative analyses and was involved in drafting the manuscript. JH was involved in the design and interpretation of experiments that were performed to measure photosynthesis rates and in drafting the manuscript. FG, RYW, RB, DDR, MGMA, and SS were majorly involved in overall experimental design and preparing the manuscript. All authors read and approved the final manuscript.

### OPEN RESEARCH BADGES

This article has earned an Open Data badge for making publicly available the digitally‐shareable data necessary to reproduce the reported results. The data is available at https://www.ncbi.nlm.nih.gov/bioproject/PRJNA612790.

## Supporting information


**Table S1**. Key raw data measured with the LI‐COR 6400XT.
**Table S2.** anova on gross photosynthesis rates.
**Table S3.** Tukey's range test on net and gross photosynthesis rates.
**Table S4.** Respiration rates in the dark.
**Table S5.** Flow cytometry‐based haploid genome size estimation.
**Table S6.** Summary statistics of raw sequencing data.
**Table S7.** Full QUAST report of nuclear assembly of *H. incana*.
**Table S8.** Comparing chloroplast assemblies of *H. incana*, *A. thaliana*, *B. rapa*, and *B. nigra*.
**Table S9.** Alignment of *H. incana* read data against the nuclear assembly.
**Table S10.** Homology groups of Brassicaceae.
**Table S11.** Homology groups containing photosynthesis‐associated genes.
**Table S12.** Genes in homology groups expanded in *H. incana*, relative to *A. thaliana*, and found at an equal or higher number of copies in *B. rapa* and *B. nigra*.
**Table S13.** Enriched GO terms in homology groups expanded in *H. incana*, relative to *A. thaliana*, and found at an equal or higher number of copies in *B. rapa* and *B. nigra*.
**Table S14.** Used genomic assemblies.
**Table S15.** *Arabidopsis thaliana* loci of genes of which expression was measured.
**Table S16.** Primer sequences.

**Figure S1**. Net CO_2_ assimilation rates as measured with the LI‐COR 6400XT.
**Figure S2.** Root tip smears of *H. incana*.
**Figure S3.** Orthologous syntenic blocks between the genomes of *H. incana* and *B. rapa*.
**Figure S4.** Normalised relative gene expression per species and light treatment for the set of multi‐copy photosynthesis‐related genes.
**Figure S5.** Normalised relative gene expression per species and light treatment for the set of single‐copy photosynthesis‐related genes.
**Figure S6.** Histogram of mismatch rates of PacBio reads aligned against the chloroplast and mitochondrial assembly of *Hirschfeldia incana*.
**Methods S1.** Pre‐processing of reads. Identifying nuclear, organellar, and contaminant reads. Assembling the chloroplast genome. Assembling the mitochondrial genome. Assembling the nuclear genome. Genome annotation.

## Data Availability

Raw sequencing data of *H. incana* can be found on the repository of the National Center for Biotechnology Information (NCBI) (BioProject ID: PRJNA612790). The genome assembly of *H. incana* has been deposited at DDBJ/ENA/GenBank under accession number JABCMI000000000. The version described in this manuscript is version JABCMI010000000. The genome assembly and annotation files, as well as a Jbrowse instance of the genome, can be accessed at https://www.bioinformatics.nl/hirschfeldia.
