# Supplementary material for: The genome sequence of Hirschfeldia incana, a new Brassicaceae model to improve photosynthetic light‐use efficiency
Source: Plant J. 2022 Nov 7;112(5):1298–315. doi: 10.1111/tpj.16005 (PMC10100226; doi:10.1111/tpj.16005)
Supplement: Supplementary file 2 — Figure S1. Net CO2 assimilation rates as measured with the LI‐COR 6400XT. Figure S2. Root tip smears of H. incana. Figure S3. Orthologous syntenic blocks between the genomes of H. incana and B. rapa. Figure S4. Normalised relative gene expression per species and light treatment for the set of multi‐copy photosynthesis‐related genes. Figure S5. Normalised relative gene expression per species and light treatment for the set of single‐copy photosynthesis‐related genes. Figure S6. Histogram of mismatch rates of PacBio reads aligned against the chloroplast and mitochondrial assembly of Hirschfeldia incana. Methods S1. Pre‐processing of reads. Identifying nuclear, organellar, and contaminant reads. Assembling the chloroplast genome. Assembling the mitochondrial genome. Assembling the nuclear genome. Genome annotation. [file TPJ-112-1298-s002.pdf]

Supplementary Figures

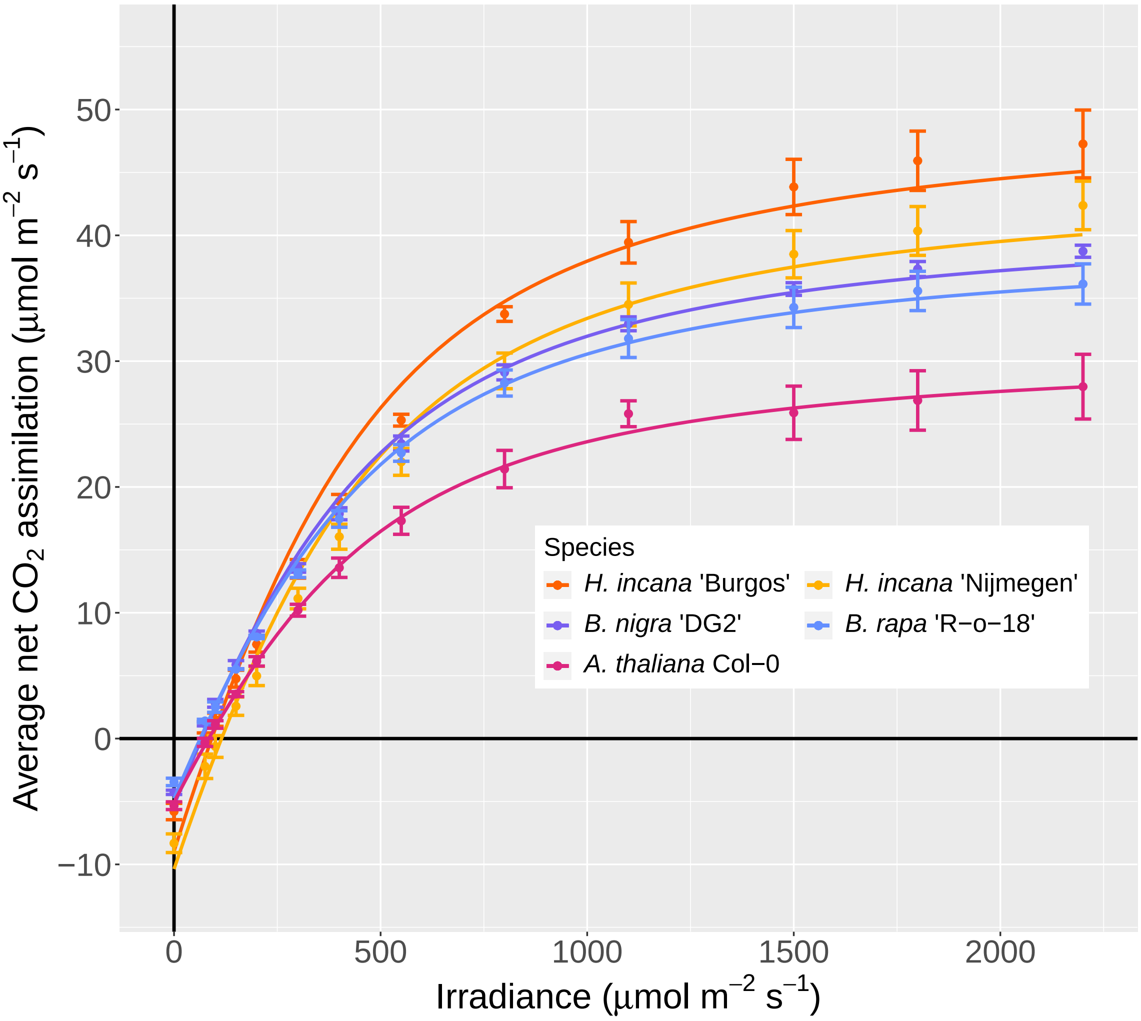

Figure S1: **Net CO_2 assimilation rates as measured with the LiCor 6400XT.** Light-response curves for *Hirschfeldia incana*, *Brassica rapa*, *Brassica nigra*, and *Arabidopsis thaliana* accessions adapted to high levels of irradiance. Each point represents the mean net CO_2 assimilation value of three (*B. rapa*) or four leaves coming from independent plants. The lines represent non-rectangular hyperbolas fitted on the raw data to estimate daytime dark respiration (R_d). Error bars represent the standard error of the means.

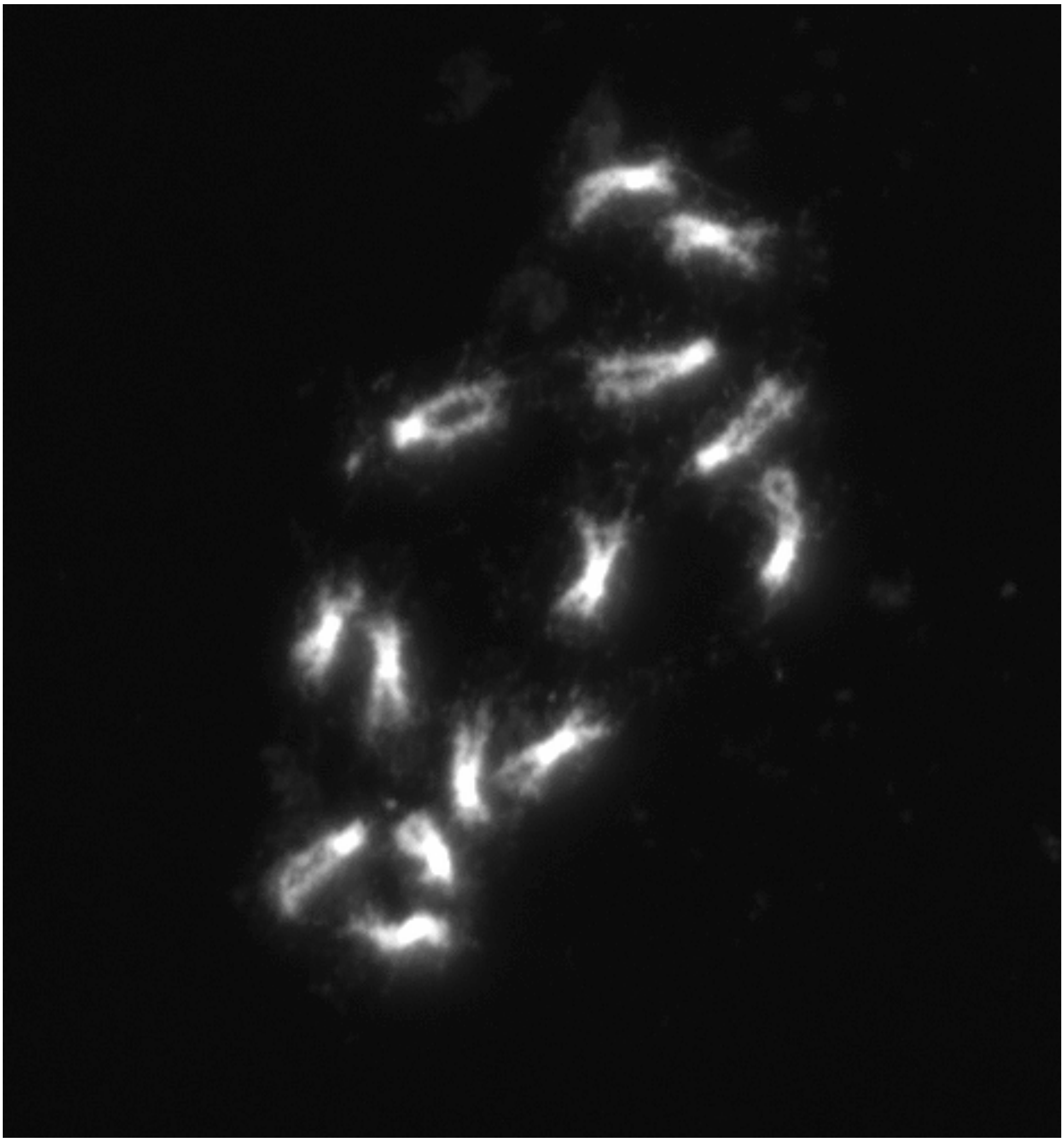

Figure S2: **Root tip smears of *Hirschfeldia incana*.** An example of 4′,6-diamidino-2-phenylindole (DAPI)-stained chromosomes from one metaphase mitotic cell. Fourteen chromosomes can be observed, meaning that *H. incana* has a haploid chromosome number of $n = 7$.

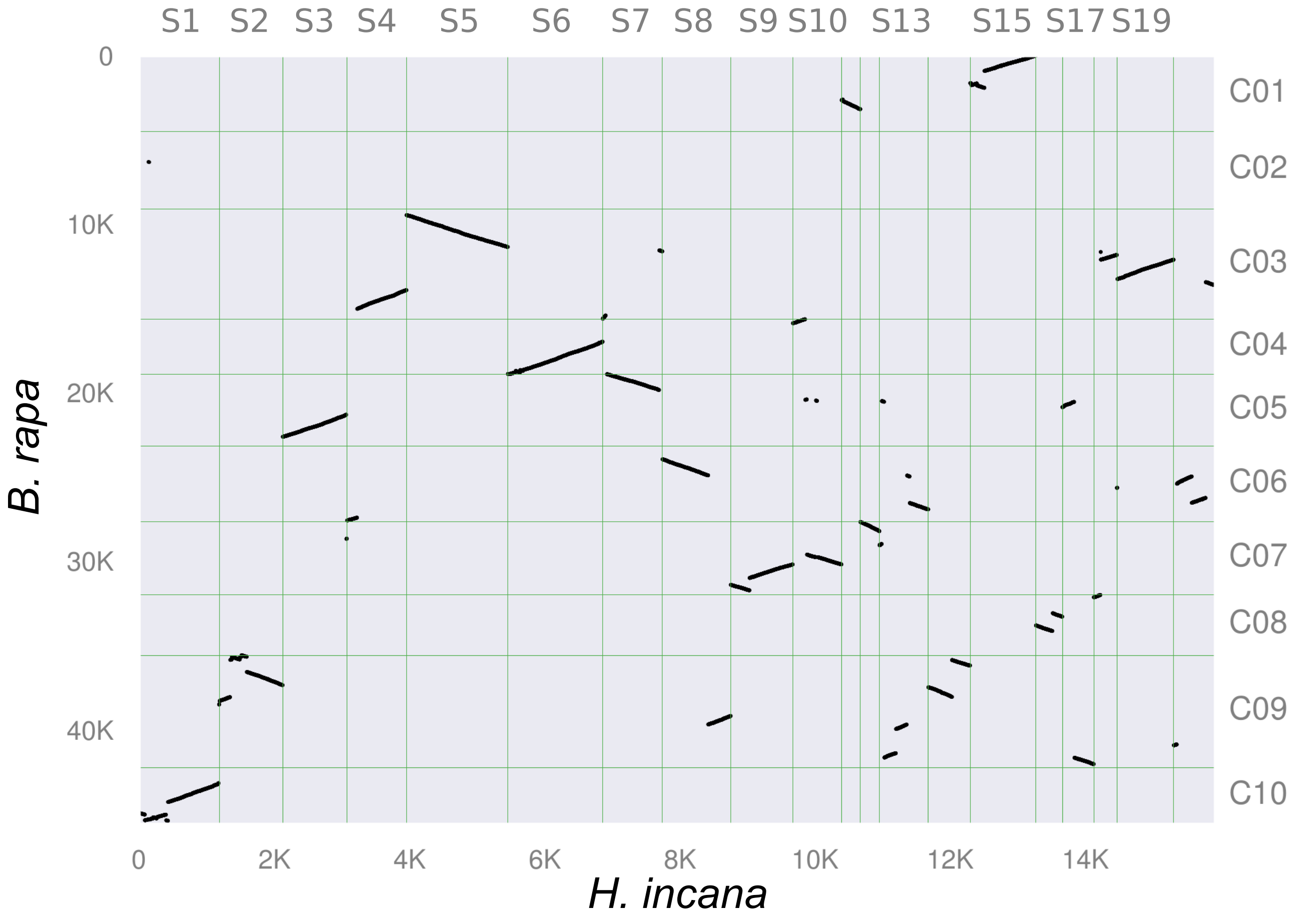

Figure S3: **Orthologous syntenic blocks between the genomes of *Hirschfeldia incana* and *Brassica rapa*.** Dots indicate pairs of syntenic orthologous that are found in the same order in both genomes according to sequence position. Only the twenty largest scaffolds of *H. incana* (43.6% of the assembly) are shown for clarity. Axes labels correspond to the total number of genes annotated on the sequences (left and bottom) and identifiers of the scaffolds (top) or chromosomes (right).

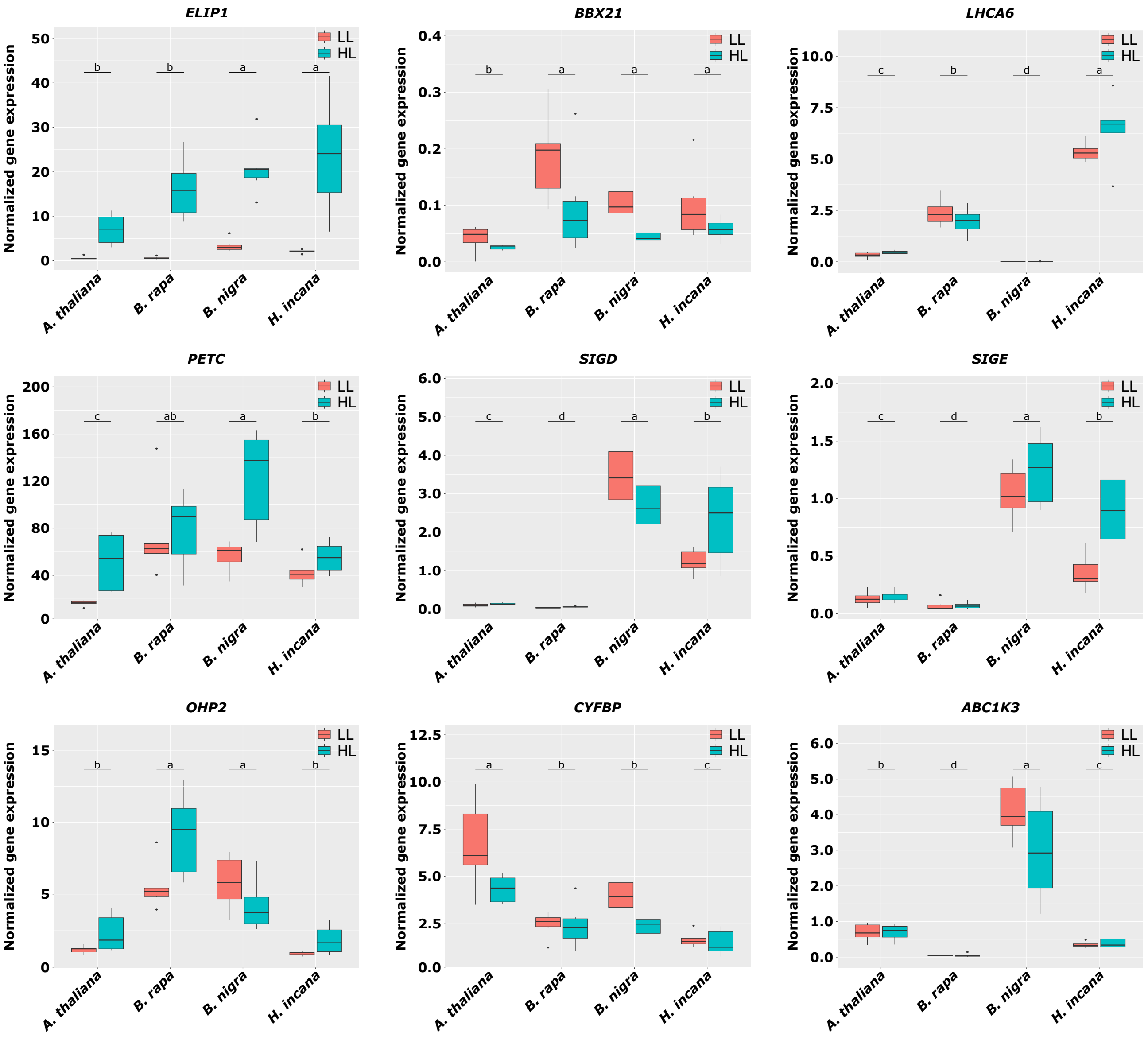

Figure S4: **Normalized relative gene expression per species and light treatment for the set of multi-copy photosynthesis-related genes.** Boxplots depict normalized gene expression levels of *Arabidopsis thaliana*, *Brassica rapa*, *Brassica nigra*, and *Hirschfeldia incana* grown in 200 µmol m^{-2} s^{-1} (LL) and 1800 µmol m^{-2} s^{-1} (HL) for genes for which there is variation in gene copy number between these species. Graph titles indicate gene names based on the *A. thaliana* gene nomenclature. Letters indicate statistical differences between species based on a two-way ANOVA and Tukey posthoc test, testing for light treatment and species as variables.

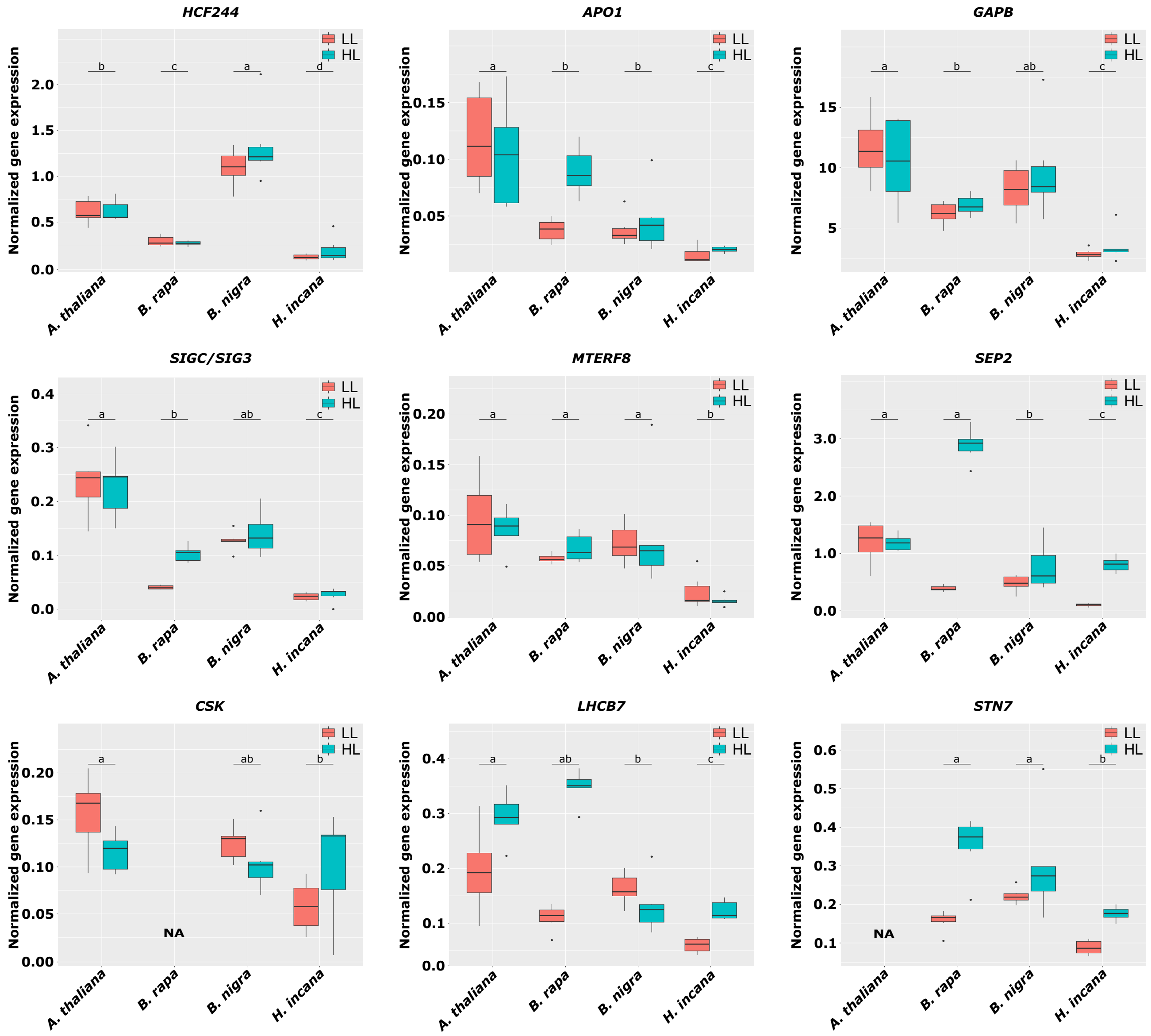

Figure S5: **Normalized relative gene expression per species and light treatment for the set of single-copy photosynthesis-related genes.** Boxplots depict normalized gene expression levels of *Arabidopsis thaliana*, *Brassica rapa*, *Brassica nigra*, and *Hirschfeldia incana* grown in 200 µmol m^{-2} s^{-1} (LL) and 1800 µmol m^{-2} s^{-1} (HL) for single-copy genes in these four species. Graph titles indicate gene names based on the *A. thaliana* gene nomenclature. Letters indicate statistical differences between species based on a two-way ANOVA and Tukey posthoc test, testing for light treatment and species as variables.

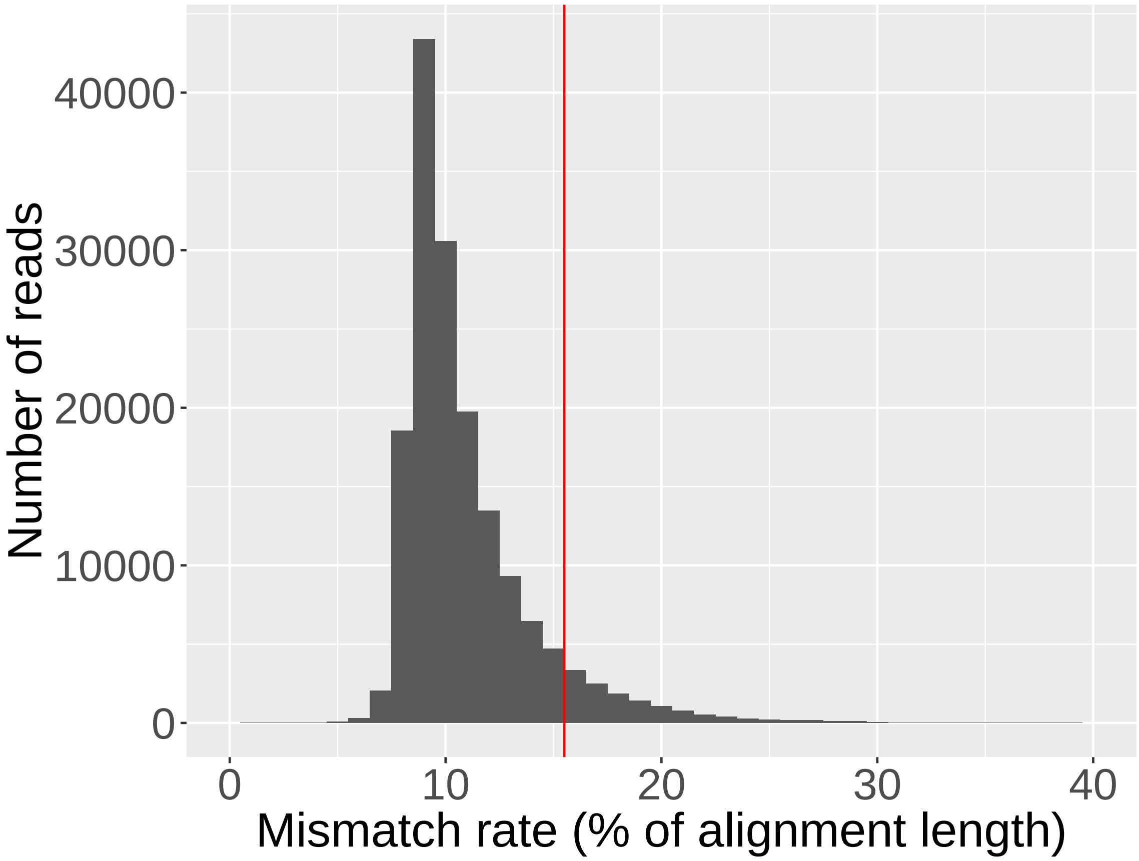

Figure S6: **Histogram of mismatch rates of PacBio reads aligned against the chloroplast and mitochondrial assembly of *Hirschfeldia incana.*** The cut-off of 15%, used to distinguish organelle-derived reads from reads originating from nuclear regions that contain organellar inserts, is depicted by the red vertical line.

Methods S1

Pre-processing of reads

PacBio

Unless specified otherwise, we used the PacBio data in its native BAM format. For downstream applications which required FASTQ format, we converted the data from BAM to FASTQ using bam2fastq (version 1.3.0) (https://github.com/PacificBiosciences/bam2fastx).

10X

Unless specified otherwise, we used the 10X data in its native FASTQ format, which includes barcodes. The barcodes were removed using Long Ranger (version 2.2.2) (https://support.10xgenomics.com/genome-exome/software/downloads/latest) (longranger basic command), if downstream applications required it.

Illumina

We trimmed Illumina data using Cutadapt (version 1.18) (Martin, 2011). Sequences were clipped from the 5' and/or the 3' ends of reads if they matched for at least 90% of their total length to one of the adapter sequences provided in the NEBNext Multiplex Oligos for Illumina (Index Primers Set 1). In addition, we trimmed bases which had a phred quality score of 30 or lower. Reads that were shorter than 100 bp after trimming were discarded. We removed duplicate Illumina read pairs using clumpify.sh of the BBTools suite (version 37.66) (https://jgi.doe.gov/data-and-tools/bbtools), allowing a maximum of three substitutions between putative duplicate pairs. The resulting deduplicated dataset was used for all downstream applications involving Illumina data.

Identifying nuclear, organellar, and contaminant reads

Identifying the origin of contigs in an initial assembly

We generated an initial genomic assembly of the PacBio data using Canu (version 1.8) (Koren et al., 2017) with the parameters genomeSize=325m and correctedErrorRate=0.040. As the PacBio data was generated from total-cellular DNA, the assembly contained contigs of mixed origin: nuclear, mitochondrial, chloroplast, or contaminant. To identify the origin of each contig, we aligned the assembly against the NCBI nucleotide (nt) database (obtained from ftp://ftp.ncbi.nih.gov/blast/db on 10-09-2019) using the command line version of blastn (version 2.6.0) (Camacho et al., 2009), retaining the best hit of each contig. In addition, we investigated the coverage of each contigs by mapping PacBio reads against the assembly using pbmm2 (https://github.com/PacificBiosciences/pbmm2), a C++ wrapper of minimap2 (Li, 2018) that can process PacBio reads in their native BAM format. Most contigs had hits against nuclear, mitochondrial, and chloroplast sequences of other plant genomes, primarily those of the Brassicaceae family. A small set of contigs had hits with genomes of contaminants such as mite and mite predator species, which act as pests or biological control, respectively. Consistent with known differences between relative copy numbers of nuclear and organellar DNA in plant species, contigs with nuclear hits generally had lower coverage (around 100x) than those with mitochondrial (900-1,100x) and chloroplast (at least 10,000x) hits. As such

large differences in coverage can obfuscate the assembly process, we used both BLAST hits and coverage information to partition PacBio reads into different bins according to their origin.

Extracting chloroplast reads

We considered contigs to represent chloroplast sequence if they had excessive coverage of PacBio reads (at least 10,000x) and a BLAST hit to a plant chloroplast sequence in the nt database. We extracted alignments of reads to such contigs with samtools view (version 1.9) (Li et al., 2009) and samtools collate, and recovered the original read sequences using bedtools bedtobam (version 2.27.1) (Quinlan & Hall, 2010). To reduce coverage before proceeding with assembly, reads were subsampled to an estimated 200-300x coverage of the chloroplast genome with seqtk sample (version 1.2-r102-dirty) (https://github.com/lh3/seqtk).

Extracting mitochondrial reads

We considered contigs to represent mitochondrial DNA if they had 900-1,100x coverage of PacBio reads and a BLAST hit to a plant mitochondrial sequence in the nt database. The contigs that fulfilled these criteria had a combined length of 1 Mb, corresponding to about four copies of the mitochondrial genome. Therefore, we estimated that the reads that aligned to these contigs corresponded to about 4,000x coverage of the complete mitochondrial genome. We extracted these alignments, recovered the original read sequences, and subsampled reads to an estimated 200-300x coverage of the mitochondrial genome, using the same commands as described in *Extracting chloroplast reads.*

Assembling the chloroplast genome

Assembly

We assembled the chloroplast reads using Flye (version 2.5-release) (Kolmogorov et al., 2019) with the parameters –genome-size 200000 and –plasmid, yielding one single contiguous sequence. The assembly was polished by running one round of Arrow (version 1.0.0) (https://github.com/PacificBiosciences/gcpp), using alignments produced by pbmm2 of all raw PacBio reads to the assembly as input. The alignments were subsampled to 200-300x coverage with Picard DownsampleSam (version 2.9.2-SNAPSHOT) (https://github.com/broadinstitute/picard) to reduce running time.

Error correction

We performed detailed manual inspection to identify and correct errors in the chloroplast assembly. To check for correct circularization, we exchanged the positions of both halves of the contig and aligned the Illumina reads to it with bwa mem (version 0.7.10-r789) (Li, 2013). We subsampled the alignments to about 200x coverage with Picard DownsampleSam and called indels using Freebayes (version v1.3.1-dirty) (Garrison & Marth, 2012) (parameters -p 1 -F 0.5 -m 50 –min-coverage 10 –read-mismatch-limit 5 –read-snp-limit 5 –limit-coverage 100) and GRIDSS (version 1.8.1-gridss) (Cameron et al., 2017) (default parameters). This analysis revealed one 31 bp insertion in the middle of the sequence (the circularisation site of the original, unmodified contig) and two homozygous 1 bp indels outside of it, which were all corrected using bcftools consensus (version 1.9) (Li, 2011).

Correcting orientation

It is conventional to report the sequence of plant chloroplasts in the following order: the long single copy (LSC) region, the first copy of the inverted repeat (IR), the short single copy (SSC) region, and finally the second copy of the inverted repeat region. We identified these regions in the assembly using the CPGAVAS2 web server (Shi et al., 2019) (accessed on September 20, 2019) and reordered the sequence of the contig accordingly to follow conventions. To check whether all regions were correctly oriented, we aligned our assembly to the chloroplast sequences of *A. thaliana*, *B. rapa*, and *B. napus* using minimap2 with parameter -ax asm20, revealing that the LSC of the chloroplast assembly of *H. incana* had been inverted relative to the LSC of the other three chloroplast assemblies. We manually inverted the LSC of the H. incana sequence to correct this error. We performed a final round of polishing with Arrow and FreeBayes/BCFtools (indels only) and repeated the check for correct circularisation, which resulted in the manual insertion of a single missing A at the end of the sequence.

Validation using an Illumina-based assembly

We generated an Illumina-based assembly of the chloroplast as an additional means of validation. Raw Illumina reads were assembled using IOGA (Bakker et al., 2016), yielding one contig containing the LSC and IR, and one contig containing the SSC. These contigs were concatenated and the reverse complement of the IR was added at the end to create a complete chloroplast sequence. The sequence was polished by aligning Illumina reads to it with Bowtie2 (Langmead & Salzberg, 2012) and using the resulting alignments as input to Pilon (Walker et al., 2014) and GapFiller (Boetzer & Pirovano, 2012) with default parameters. Any remaining stretches of Ns were filled by aligning the assembly to the *A. thaliana* chloroplast sequence with blastn and manually replacing the Ns by the *A. thaliana* sequence that aligned to them. Both halves of the assembly were exchanged to check whether the sequence properly circularized, resulting in the removal of 27 bp of sequence that overlapped between both ends. The final Illumina-based assembly was identical to the one based on PacBio reads, suggesting that both correctly represent the chloroplast sequence of *H. incana.*

Annotation

The chloroplast assembly was annotated using the cpGAVAS2 workflow (Shi et al., 2019) with default parameters and the 43 RNA-seq curated plastomes as reference set.

Assembling the mitochondrial genome

Assembly

We assembled mitochondrial reads using Canu with parameters genomeSize=250k and corrected-ErrorRate=0.040, yielding five contigs. Inspecting the assembly graph produced by Canu with Bandage (version 0.8.1) (Wick et al., 2015) revealed that two contigs formed a circular sequence, while the other three contigs corresponded to "dead-ends". The presence of multiple possible assembly paths is consistent with previous studies that have shown that plant mitochondrial DNA typically consists of a "master circle" confirmation containing the full mitochondrial sequence and multiple alternative subgenomic circular and linear conformations generated by repeat-induced recombination (Sloan, 2013). As it is conventional to report the "master circle" sequence (Sloan et

al., 2018), we mapped all the reads that were used as input to the five contigs with minimap2 (parameter -ax map-pb) and only retained those that aligned to the two contigs forming the circular sequence. Running Canu (parameters: genomeSize=250k, correctedErrorRate=0.040) on these reads produced one single contig representing the “master circle” conformation.

Error correction

We ran Circlator (–b2r_discard_unmapped) (version 1.5.5-docker2) (Hunt et al., 2015) to correct any errors regarding circularization, using all mitochondrion-derived reads as input. We used the same set of reads to call structural variants with NGMLR (version 0.2.7) and Sniffles (version 1.0.9) (Sedlazeck et al., 2018) (parameters: -s 20). No structural variants were reported by this analysis, suggesting that the assembly was free of large misassemblies. We polished the assembly using two rounds of Arrow, aligning raw PacBio reads with pbmm2 and subsampling alignments to about 200-300x coverage with Picard DownsampleSam at each round. We manually assessed whether the assembly properly circularized by exchanging both halves of the sequence and mapping Illumina reads to it using bwa mem with default parameters. The chloroplast assembly of *H. incana* was included in the alignment procedure to prevent cross-mapping of chloroplast-derived reads. We used the alignments to call SNPs and indels with FreeBayes, resulting in a small set of small variants. However, all of them were located in regions which had relatively higher coverage than the rest of the genome. The nuclear genome of plants is known to contain inserted DNA that is highly similar to mitochondrial sequence and we suspect that the cross-mapping of such reads is what causes this rise in coverage. Thus, we consider any variants called in these regions to be incorrect. This result, combined with the absence of large structural variation based on mitochondrion-derived reads, suggests that our mitochondrial assembly is both accurate and complete.

Assembling the nuclear genome

Extracting nuclear reads

We attempted to remove non-nuclear reads from the PacBio data by mapping it with pbmm2 against the chloroplast and mitochondrial assembly (both duplicated to catch reads that span both ends), the reference genomes of any contaminants to which the contigs of the initial assembly had a BLAST hit, and contigs that had a BLAST hit with a contaminant. Unmapped reads in the output file were retained using the parameter –unmapped. A custom Python script based on the pysam library (https://github.com/pysam-developers/pysam) was used to extract all putative nuclear reads from the alignments. We considered reads to be of nuclear origin if they fulfilled any of the following conditions: the read was unmapped, the read had an alignment which was clipped by more than 5% of its length, or the read had an alignment which consisted for over 15% of its length out of mismatches. This latter threshold was chosen based on a histogram of the mismatch rate of all aligned reads, which showed a peak at about 10% (Figure S6), consistent with the known sequencing error rate of the PacBio Sequel platform (Logsdon et al., 2020). Given that the organelles were sequenced at much higher depths than the nuclear genome, we considered reads which had such a mismatch rate to be derived from organelles and any reads that had a mismatch rate higher than this peak (over 15%) to be nuclear-derived.

Assembly

We assembled nuclear reads using Canu (parameters: genomeSize=325m, correctedErrorRate=0.040). To remove misassemblies, we identified and broke putative misassemblies of the Canu assembly with Tigmint (version 1.1.2) (Jackman et al., 2018), which uses alignments of 10X reads as input. Alignments were produced using bwa mem with default parameters on 10X data processed by Long Ranger. Besides the assembly produced by Canu, the organellar assemblies and reference genomes of possible contaminants were included in the alignment procedure to prevent organellar and contaminant reads from introducing false misassemblies. Alignments were sorted by barcode using samtools sort (parameter -tBX).

Quality control

We assessed the quality of the assembly through several measures. We determined the contiguity of the assembly using QUAST (version 5.0.2) (Gurevich et al., 2013). To estimate the completeness of the genic space, we aligned RNA-seq reads to the assembly with HISAT2 (version 2.1.0) (Kim et al., 2019). We executed BUSCO (version 3.0.2) (Simão et al., 2015) to determine how well the assembly covered the embryophyta_odb9 database of conserved plant orthologs. To identify erroneously assembled contigs, we mapped the putative nuclear-derived PacBio reads to it with minimap2 (-ax map-pb) and computed the read depth of each contig with mosdepth (version 0.2.6) (Pedersen & Quinlan, 2018). Finally, we identified possible contaminant contigs by aligning the Canu assembly against the nt database with blastn (-max_target_seqs 1 -max_hsps 1 -evalue 1e-25).

Filtering contigs

The results of the quality control step were used to design a set of filters for obtaining contigs that are expected to be reasonably accurate. We kept contigs that were covered for at least 90% of their length by at least one PacBio read, if they had no BLAST hit with a non-plant derived sequence, and fulfilled any of the following conditions: having a median read depth of at least 30X, containing a BUSCO, or having RNA-seq coverage.

Removing duplicate haplotigs and scaffolding

We purged alternative haplotigs from the assembly using purge_dups (version 1.0.0) (Guan et al., 2020) with default parameters. The purged assembly was scaffolded with ARKS (version 1.0.4) (Coombe et al., 2018) (parameters c=8 m=8-10000), a tool which infers linkage between contigs using 10X data.

Gap-filling and polishing

After performing scaffolding, we filled gaps and polished the assembly with Arrow, using alignments of raw PacBio reads as input. The organellar assemblies and earlier identified contaminant and filtered contigs were included in the reference file during alignment to prevent reads derived from these sequences from obfuscating the polishing step. To remove any remaining single nucleotide level errors, we aligned 10X reads to the polished assembly using bwa mem, again including organellar, contaminant and filtered sequences in the reference file to prevent cross-mapping. We called variants

in the assembly using FreeBayes (-p 2 -F 0.5 -m 50 –min-coverage 10 –read-mismatch-limit 5 –read-snp-limit 5 –limit-coverage 100) and corrected all indels that were well supported using bcftools consensus ('QUAL>1 && TYPE="indel" && (GT="AA" || GT="Aa")').

Filtering scaffolds

We used the same quality control steps as described in the *Quality control* section to assess the accuracy of all polished scaffolds. As an additional means of validation, we mapped 10X reads and Illumina to the polished scaffolds, organellar, contaminant and filtered sequences using respectively Long Ranger (longranger align) and bwa mem with default parameters. We designed another set of filters to only retain scaffolds which we deemed to be highly accurate. Scaffolds were kept if they contained a BUSCO or at least 10000 bp of RNA-seq coverage. Of the set that remained, we excluded scaffolds of which fewer than 90% of the length had a coverage of at least 6x based on Illumina reads, no BLAST hit with a plant sequence of the nt database, or fewer than 30x and 25x median coverage of PacBio on Illumina reads, respectively.

Removing sequences masked during duplicate haplotig removal

The assembly contained regions of 23 Ns, which were introduced by purge_dups while removing duplicated haplotig sequences. Although we were not able to ascertain that all sequences connected by these regions should be linked, there was evidence that some of the links were correct as breaking the scaffolds at these regions resulted in a loss of complete BUSCOs. Therefore, we decided to keep most of these regions in the assembly, only removing those that were present within 5 kb of scaffold ends, along with the associated sequences present at those ends.

Removing organellar sequences

To check for organellar sequences, we aligned the nuclear assembly against the mitochondrial and chloroplast assembly using blastn (-max_target_seqs 10 -max_hsps 10 -evalue 700). This revealed four nuclear regions which had near 100% identity with regions of the mitochondrial assembly and had significantly higher coverage of Illumina reads compared to the genomic average. We considered these regions to be mitochondrial and removed them from the nuclear assembly.

Genome annotation

Repeat annotation

RepeatModeler2 (Flynn et al., 2020) (run with the -LTRstruct parameter) was used to generate a de novo library of repetitive elements for the *H. incana* nuclear genome, after which RepeatMasker (Smit et al., 2015) was used to soft-mask those elements. Both tools were called through TETools v1.1 (https://github.com/Dfam-consortium/TETools).

Gene annotation

We annotated putative protein-coding genes in the nuclear assembly using EvidenceModeler (commit 5d9425a, https://github.com/EVidenceModeler/EVidenceModeler) (Haas et al., 2008), which performs a weighted combination of de novo gene predictions, protein alignments, and transcript

assemblies. The input for EvidenceModeler was generated as follows. We aligned RNA-seq reads to the nuclear and organellar assemblies of *H. incana*, as well as against filtered contigs and contigs of possible contaminants. Reads that mapped against the nuclear assembly were extracted and used along with the masked nuclear genomic assembly as input to BRAKER (version 2.1.5) (Hoff et al., 2016), which internally calls Genemark-ET (in RNA-seq guided mode) (Lomsadze et al., 2014) and Augustus (Stanke et al., 2008) to predict de novo gene models. We assembled transcripts out of the RNA-seq read alignments using Stringtie2 (version 2.1.1) (Kovaka et al., 2019) and detected ORFs in these transcripts using transdecoder (version 5.5.0) (github.com/TransDecoder/TransDecoder), both with default parameters. We obtained protein sets of *A. thaliana* (www.arabidopsis.org/download_files/Proteins/Araport11_protein_lists/Araport11_genes.201606.pep.fasta.gz), *B. rapa* (brassicadb.org/brad/datasets/pub/Genomes/Brassica_rapa/V3.0/Brapa_genome_v3.0_pep.fasta.gz), *B. nigra* (longest isoforms of the Ni100 annotation, translated with gffread), and the Viridiplantae clade (SwissProt set only: www.uniprot.org/uniprot/?query=reviewed:yes%20taxonomy:33090). All four sets of proteins were aligned to the *H. incana* nuclear genome using GenomeThreader (version 1.7.3) (Gremme et al., 2005) (parameters: -species arabidopsis -gff3out -skipalignmentout).

The output of the two gene predictors, the four sets of protein alignments, and the ORFs predicted by transdecoder were given as input to EvidenceModeler to generate consensus gene models. These models were then processed with PASA (version 2.4.1) (Haas et al., 2003) to add UTRs and define alternative transcripts for each gene. PASA was ran by first aligning the transcripts assembled by Stringtie2 to the *H. incana* nuclear genome with GMAP and generating maximum alignment assemblies out of the alignments, followed by two rounds of updates to the EvidenceModeler consensus models. The final output of PASA was used for further filtering steps.

Filtering gene annotation

We filtered transcripts based on homology with proteins with close relatives and support from RNA-seq reads. We extracted the longest isoforms of the annotated genes in *A. thaliana*, *B. rapa* (v3.0 assembly), and *B. nigra* (Ni100 v2 assembly) using AGAT (version 0.2.3) (github.com/NBISweden/AGAT), converted them into protein sequences using gffread (v0.11.8) (https://github.com/gpertea/gffread), and clustered them with the protein products of *H. incana* into orthogroups using OrthoFinder (version 2.3.11) (Emms & Kelly, 2019). The *A. thaliana* annotation used was a combination of the TAIR10 genomic annotation of Ensembl Plants (release version 40) and the annotation of an improved A. thaliana mitochondrial assembly (BK010421.1).

We extracted the exons of the *H. incana* annotation in BED format and used mosdepth (version 0.2.9) (ran with the parameter -T 1) to determine which exons were supported by at least one RNA-seq read. We extracted intron-spanning reads of the RNA-seq alignments and introns from the *H. incana* annotation using custom Python scripts and intersected them with bedtools intersect to compute the support for each intron.

Transcripts were then filtered using the following the criteria:

- All introns supported by at least one RNA-seq read, at least 85% reciprocal overlap with a transcript assembly produced by Stringtie2
- Overlap with a protein alignment of *A. thaliana*; *B. rapa*, or the Viridiplantae SwissProt dataset for at least 85% of the length of the protein alignment and at least 25% of the length

of the transcript (the length threshold for the transcript is less strict, because a protein alignment lacks UTRs);

- Has a homolog with an *A. thaliana* gene involved in photosynthesis.

Transcripts which fulfilled at least one of these three criteria were put in the "accepted" set and those that did not were put in the "rejected" set. As a final filtering step, we removed transcripts of the "accepted" set which overlapped for over 85% of their length with repetitive elements identified by RepeatMasker, as these are likely to be involved with transposable elements.

A. thaliana genes were considered to be involved in photosynthesis, if they fulfilled one of the following conditions:

- Assigned one of the following GO terms: "photosynthesis", "electron transporter, transferring electrons within the cyclic electron transport pathway of photosynthesis activity", or "electron transporter, transferring electrons within the noncyclic electron transport pathway of photosynthesis activity";
- Included in the KEGG pathways ath00195 (Photosynthesis), ath00710 (Carbon fixation in photosynthetic organisms), and ath00196 (Photosynthesis - Antenna Proteins);
- Protein products have been assigned the keyword "Photosynthesis" in the Swiss-Prot database.

Assessing completeness through protein alignments of *B. rapa*

The longest isoforms of *B. rapa* genes were obtained using AGAT and translated to proteins with gffread. Translated proteins were aligned to the *H. incana* assembly using GenomeThreader (parameters: -species arabidopsis -gff3out -skipalignmentout), that reports matches if proteins align to the assembly for at least 50% of their length. 31002 (67%) of the *B. rapa* proteins could be aligned to the *H. incana* assembly in this manner, resulting in a total of 37283 alignments.

References

Bakker, F. T., Lei, D., Yu, J., Mohammadin, S., Wei, Z., van de Kerke, S., Gravendeel, B., Nieuwenhuis, M., Staats, M., Alquezar-Planas, D. E., & Holmer, R. (2016). Herbarium genomics: plastome sequence assembly from a range of herbarium specimens using an Iterative Organelle Genome Assembly pipeline. *Biological Journal of the Linnean Society*, *117*(1), 33–43.

Boetzer, M., & Pirovano, W. (2012). Toward almost closed genomes with GapFiller. *Genome Biology*, *13*(6), R56.

Camacho, C., Coulouris, G., Avagyan, V., Ma, N., Papadopoulos, J., Bealer, K., & Madden, T. L. (2009). BLAST+: architecture and applications. *BMC Bioinformatics*, *10*(1), 421.

Cameron, D. L., Schröder, J., Penington, J. S., Do, H., Molania, R., Dobrovic, A., Speed, T. P., & Papenfuss, A. T. (2017). GRIDSS: sensitive and specific genomic rearrangement detection using positional de Bruijn graph assembly. *Genome Research*, *27*(12), 2050–2060.

Coombe, L., Zhang, J., Vandervalk, B. P., Chu, J., Jackman, S. D., Birol, I., & Warren, R. L. (2018). ARKS: chromosome-scale scaffolding of human genome drafts with linked read kmers. *BMC Bioinformatics*, *19*(1), 1–10.

Emms, D. M., & Kelly, S. (2019). Orthofinder: Phylogenetic orthology inference for comparative genomics. *Genome Biology*, *20*(1), 1–14.

Flynn, J. M., Hubley, R., Goubert, C., Rosen, J., Clark, A. G., Feschotte, C., & Smit, A. F. (2020). Repeatmodeler2 for automated genomic discovery of transposable element families. *Proceedings of the National Academy of Sciences*, *117*(17), 9451–9457.

Garrison, E., & Marth, G. (2012). Haplotype-based variant detection from short-read sequencing. *arXiv*. Preprint at https://arxiv.org/abs/1207.3907.

Gremme, G., Brendel, V., Sparks, M. E., & Kurtz, S. (2005). Engineering a software tool for gene structure prediction in higher organisms. *Information and Software Technology*, *47*(15), 965–978.

Guan, D., McCarthy, S. A., Wood, J., Howe, K., Wang, Y., & Durbin, R. (2020). Identifying and removing haplotypic duplication in primary genome assemblies. *Bioinformatics*, *36*(9), 2896–2898.

Gurevich, A., Saveliev, V., Vyahhi, N., & Tesler, G. (2013). QUAST: Quality assessment tool for genome assemblies. *Bioinformatics*, *29*(8), 1072–1075.

Haas, B. J., Delcher, A. L., Mount, S. M., Wortman, J. R., Smith Jr, R. K., Hannick, L. I., Maiti, R., Ronning, C. M., Rusch, D. B., Town, C. D., et al. (2003). Improving the *Arabidopsis* genome annotation using maximal transcript alignment assemblies. *Nucleic Acids Research*, *31*(19), 5654–5666.

Haas, B. J., Salzberg, S. L., Zhu, W., Pertea, M., Allen, J. E., Orvis, J., White, O., Buell, C. R., & Wortman, J. R. (2008). Automated eukaryotic gene structure annotation using EVidenceModeler and the Program to Assemble Spliced Alignments. *Genome Biology*, *9*(1), R7.

Hoff, K. J., Lange, S., Lomsadze, A., Borodovsky, M., & Stanke, M. (2016). BRAKER1: unsupervised RNA-Seq-based genome annotation with GeneMark-ET and AUGUSTUS. *Bioinformatics*, *32*(5), 767–769.

Hunt, M., De Silva, N., Otto, T. D., Parkhill, J., Keane, J. A., & Harris, S. R. (2015). Circlator: Automated circularization of genome assemblies using long sequencing reads. *Genome Biology*, *16*(1), 1–10.

Jackman, S. D., Coombe, L., Chu, J., Warren, R. L., Vandervalk, B. P., Yeo, S., Xue, Z., Mohamadi, H., Bohlmann, J., Jones, S. J., et al. (2018). Tigmint: Correcting assembly errors using linked reads from large molecules. *BMC Bioinformatics*, *19*(1), 1–10.

Kim, D., Paggi, J. M., Park, C., Bennett, C., & Salzberg, S. L. (2019). Graph-based genome alignment and genotyping with HISAT2 and HISAT-genotype. *Nature Biotechnology*, *37*(8), 907–915.

Kolmogorov, M., Yuan, J., Lin, Y., & Pevzner, P. A. (2019). Assembly of long, error-prone reads using repeat graphs. *Nature Biotechnology*, *37*(5), 540–546.

Koren, S., Walenz, B. P., Berlin, K., Miller, J. R., Bergman, N. H., & Phillippy, A. M. (2017). Canu: Scalable and accurate long-read assembly via adaptive k-mer weighting and repeat separation. *Genome Research*, *27*(5), 722–736.

Kovaka, S., Zimin, A. V., Pertea, G. M., Razaghi, R., Salzberg, S. L., & Pertea, M. (2019). Transcriptome assembly from long-read RNA-seq alignments with StringTie2. *Genome Biology*, *20*(1), 1–13.

Langmead, B., & Salzberg, S. L. (2012). Fast gapped-read alignment with Bowtie 2. *Nature Methods*, *9*(4), 357.

Li, H. (2011). A statistical framework for SNP calling, mutation discovery, association mapping and population genetical parameter estimation from sequencing data. *Bioinformatics*, *27*(21), 2987–2993.

Li, H. (2013). Aligning sequence reads, clone sequences and assembly contigs with BWA-MEM. *arXiv*. Preprint at http://arxiv.org/abs/1207.3907.

Li, H. (2018). Minimap2: Pairwise alignment for nucleotide sequences. *Bioinformatics*, *34*(18), 3094–3100.

Li, H., Handsaker, B., Wysoker, A., Fennell, T., Ruan, J., Homer, N., Marth, G., Abecasis, G., & Durbin, R. (2009). The sequence alignment/map format and SAMtools. *Bioinformatics*, *25*(16), 2078–2079.

Logsdon, G. A., Vollger, M. R., & Eichler, E. E. (2020). Long-read human genome sequencing and its applications. *Nature Reviews Genetics*, 1–18.

Lomsadze, A., Burns, P. D., & Borodovsky, M. (2014). Integration of mapped RNA-Seq reads into automatic training of eukaryotic gene finding algorithm. *Nucleic Acids Research*, *42*(15), e119–e119.

Martin, M. (2011). Cutadapt removes adapter sequences from high-throughput sequencing reads. *EMBnet.journal*, *17*(1), 10–12.

Pedersen, B. S., & Quinlan, A. R. (2018). Mosdepth: Quick coverage calculation for genomes and exomes. *Bioinformatics*, *34*(5), 867–868.

Quinlan, A. R., & Hall, I. M. (2010). BEDtools: A flexible suite of utilities for comparing genomic features. *Bioinformatics*, *26*(6), 841–842.

Sedlazeck, F. J., Rescheneder, P., Smolka, M., Fang, H., Nattestad, M., von Haeseler, A., & Schatz, M. C. (2018). Accurate detection of complex structural variations using single-molecule sequencing. *Nature Methods*, *15*(6), 461.

Shi, L., Chen, H., Jiang, M., Wang, L., Wu, X., Huang, L., & Liu, C. (2019). CPGAVAS2, an integrated plastome sequence annotator and analyzer. *Nucleic Acids Research*, *47*(W1), W65–W73.

Simão, F. A., Waterhouse, R. M., Ioannidis, P., Kriventseva, E. V., & Zdobnov, E. M. (2015). BUSCO: assessing genome assembly and annotation completeness with single-copy orthologs. *Bioinformatics*, *31*(19), 3210–3212.

Sloan, D. B. (2013). One ring to rule them all? Genome sequencing provides new insights into the 'master circle' model of plant mitochondrial DNA structure. *New Phytologist*, *200*(4), 978–985.

Sloan, D. B., Wu, Z., & Sharbrough, J. (2018). Correction of persistent errors in Arabidopsis reference mitochondrial genomes. *The Plant Cell*, *30*, 525–527.

Smit, A. F., Hubley, R., & Green, P. (2015). *Repeatmasker open-4.0.* http://www.repeatmasker.org 2013-2015

Stanke, M., Diekhans, M., Baertsch, R., & Haussler, D. (2008). Using native and syntenically mapped cDNA alignments to improve de novo gene finding. *Bioinformatics*, *24*(5), 637–644.

Walker, B. J., Abeel, T., Shea, T., Priest, M., Abouelliel, A., Sakthikumar, S., Cuomo, C. A., Zeng, Q., Wortman, J., Young, S. K., et al. (2014). Pilon: an integrated tool for comprehensive microbial variant detection and genome assembly improvement. *PloS One*, *9*(11), e112963.

Wick, R. R., Schultz, M. B., Zobel, J., & Holt, K. E. (2015). Bandage: Interactive visualization of de novo genome assemblies. *Bioinformatics*, *31*(20), 3350–3352.
